# Unraveling the cGAS/STING signaling mechanism: impact on glycerolipid metabolism and diseases

**DOI:** 10.3389/fmed.2024.1512916

**Published:** 2024-11-28

**Authors:** Jie Su, Fuyu Cheng, Wei Yuan

**Affiliations:** ^1^Department of Cardiology, Hospital of Jiangsu University, Zhenjiang, China; ^2^The British Heart Foundation Centre of Excellence, St Thomas’ Hospital, School of Cardiovascular and Metabolic Medicine and Sciences, King’s College London, The Rayne Institute, London, United Kingdom; ^3^School of Engineering and Material Sciences, Digital Environment Research Institute, Queen Mary University of London, London, United Kingdom

**Keywords:** cGAS/STING pathway, inflammation, immune response, glycerolipid metabolism, cardiovascular disease

## Abstract

The cyclic GMP-AMP synthase (cGAS) and its downstream effector, the stimulator of interferon genes (STING), are crucial components of the innate immune response, traditionally recognized for their role in detecting cytosolic DNA from pathogens and damaged host cells. However, recent research indicates that the cGAS-STING pathway also significantly impacts metabolic processes, particularly glycerolipid metabolism. Glycerolipids are essential for energy storage and cellular membrane integrity, and their dysregulation is linked to metabolic disorders such as obesity, insulin resistance, and non-alcoholic fatty liver disease (NAFLD). Both cGAS and STING are expressed in various metabolic tissues, suggesting a potential role in lipid homeostasis. Chronic activation of the cGAS-STING pathway may promote inflammatory states that exacerbate insulin resistance and lipid accumulation, forming a feedback loop of metabolic dysfunction. This review explores the emerging relationship between cGAS/STING signaling and glycerolipid metabolism, discussing the mechanisms through which this pathway influences lipid regulation and the potential for therapeutic interventions. By integrating insights from immunology and metabolism, we aim to provide a comprehensive understanding of how the cGAS-STING axis may serve as a novel target for addressing metabolic disorders and enhancing metabolic health outcomes.

## 1 Introduction

The cyclic GMP-AMP synthase (cGAS) serves as a vital sensor for cytosolic DNA, initiating a critical immune response against both pathogenic invasion and cellular damage. When DNA is improperly localized in the cytosol, cGAS catalyzes the synthesis of cyclic GMP-AMP (cGAMP) from ATP and GTP. This second messenger subsequently activates the stimulator of interferon genes (STING) pathway, leading to the production of type I interferons (IFN-1) and other pro-inflammatory cytokines (1, 2). Although the cGAS-STING signaling pathway is predominantly recognized for its role in innate immunity, recent research highlights its broader biological functions that extend into various physiological contexts, including metabolism (3, 4).

Emerging evidence suggests that the cGAS-STING pathway is intricately linked to glycerolipid metabolism, a key process in maintaining cellular energy balance and membrane integrity (5–7). Glycerolipids, which encompass triglycerides and phospholipids, are not only critical for energy storage but also play essential roles in cellular signaling and membrane dynamics. Dysregulation of glycerolipid metabolism is increasingly associated with metabolic disorders such as obesity, insulin resistance, and non-alcoholic fatty liver disease (NAFLD) (8). These conditions are characterized by imbalances in lipid synthesis and degradation, often exacerbated by inflammatory processes.

Both cGAS and STING exhibit expression patterns in various metabolic tissues, including adipose tissue, liver, and muscle, suggesting that they may contribute to the regulation of lipid metabolism (9–11). Human protein Atlas analysis has shown that cGAS is present in key metabolic organs, indicating a potential role in sensing cellular stress or damage within these tissues.^1^ Conversely, the expression of STING is more selective, predominantly found in immune tissues, but also detected in metabolic tissues, albeit at lower levels.^2^ This discrepancy prompts further investigation into how these proteins may influence metabolic processes, especially given their known roles in mediating inflammatory responses.

The interplay between cGAS/STING signaling and glycerolipid metabolism may create a complex feedback loop, where chronic activation of this pathway can lead to a heightened inflammatory state, contributing to the development of insulin resistance and fat accumulation. For instance, pro-inflammatory cytokines produced as a result of cGAS-STING activation can interfere with insulin signaling pathways, promoting a state of metabolic dysregulation. Furthermore, the association of cGAS with lipid droplets in adipocytes suggests that it may play a direct role in modulating lipid storage and mobilization, linking immune signaling with energy homeostasis.

Despite the progress made in understanding the role of cGAS and STING in immune contexts, their contributions to metabolic regulation remain underexplored. There is a pressing need to investigate how the cGAS-STING pathway influences various aspects of glycerolipid metabolism, including lipid synthesis, storage, and breakdown. Additionally, understanding the molecular mechanisms underlying these interactions could reveal new therapeutic targets for addressing metabolic diseases.

This review aims to synthesize current findings regarding the cGAS-STING signaling pathway, emphasizing its role in glycerolipid metabolism and its implications for metabolic health. By bridging the fields of immunology and metabolism, we seek to highlight the potential of targeting the cGAS-STING axis as a novel therapeutic approach for metabolic disorders. Through a comprehensive examination of the existing literature, we aim to elucidate the critical functions of this signaling pathway in regulating metabolism, ultimately contributing to a better understanding of the interplay between immune responses and metabolic health.

## 2 cGAS/STING signaling pathway

**Figure 1:**
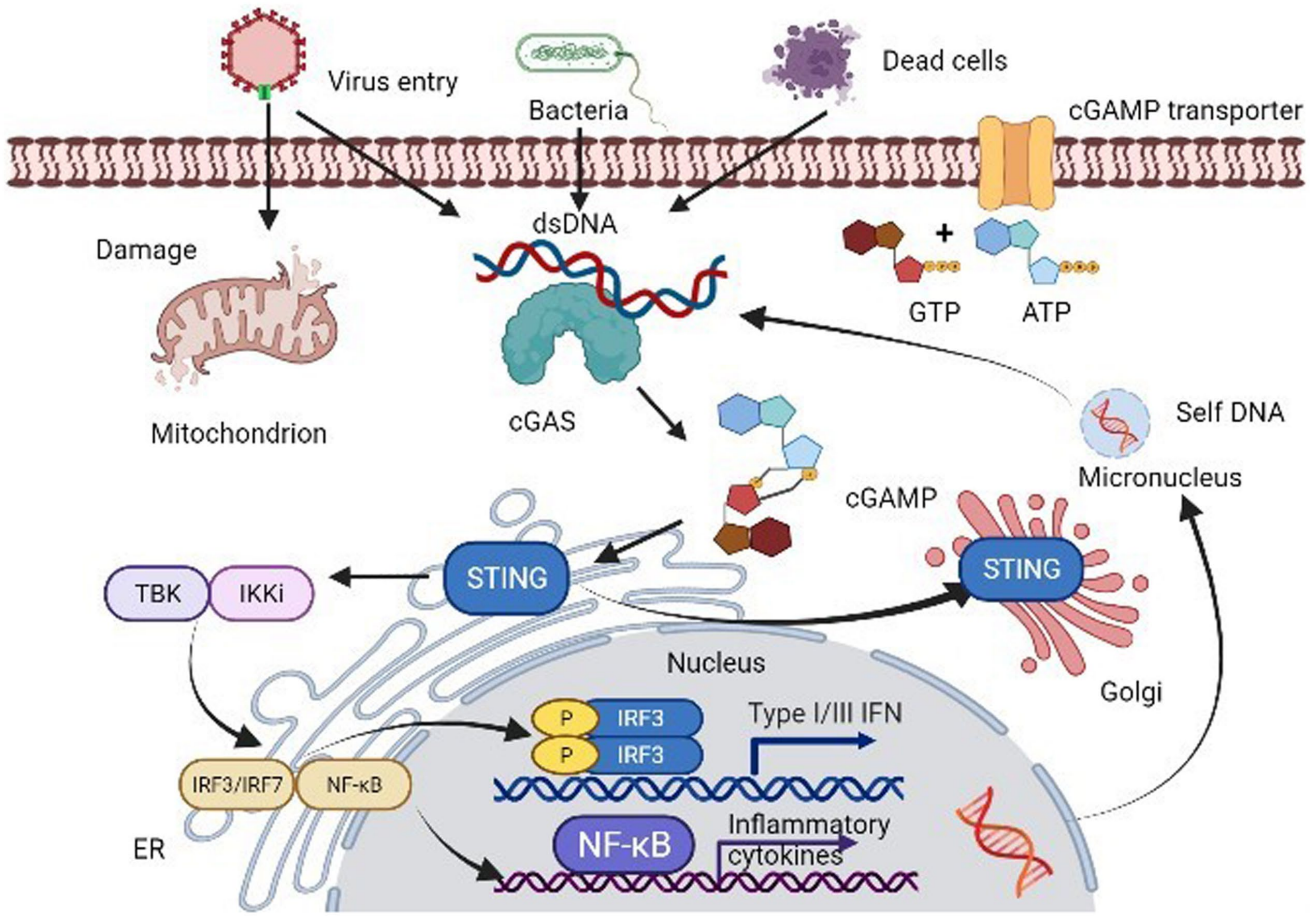
The cGAS-STING signaling pathway. cGAS not only recognizes cytoplasmic self-DNA but also a large variety of DNA-containing pathogens, such as DNA viruses and bacteria. Upon binding to dsDNA, activated cGAS produces 2′3′-cGAMP, which binds to and activates downstream STING, causing its translocation from the ER membrane to the Golgi apparatus. The translocation of STING subsequently allows itself to recruit and activate TBK1 and IRF3 via a phosphorylation dependent-mechanism. The active IRF3 dimer translocations to the nucleus and activates transcription of IFN-1 genes. In addition, activated STING triggers NF-κB and MAPK pathway activation.

The innate immune system serves as the primary defense mechanism against pathogens, employing a variety of strategies to recognize and combat infections. A key aspect of this response is the detection of pathogen-derived DNA in the cytosol, primarily mediated by the enzyme cGAS. Upon encountering double-stranded DNA (dsDNA), cGAS becomes activated and catalyzes the synthesis of the second messenger cGAMP. This molecule interacts with STING, facilitating STING’s translocation from the endoplasmic reticulum (ER) to the ER-Golgi intermediate compartment and ultimately to the Golgi apparatus. This translocation is crucial for engaging downstream signaling cascades that lead to the activation of the TANK binding kinase 1 (TBK1) and interferon regulatory factor 3 (IRF3), which drive IFN-1 expression (2). The well-recognized cGAS/STING signaling pathway is as Figure 1.

The cGAS-STING pathway is not only a first line of defense against infections but also a mediator of sterile inflammation in response to self-DNA, such as mitochondrial DNA (mtDNA) and nuclear DNA that become aberrantly localized in the cytosol due to cellular stress, genomic instability, or DNA damage. This inappropriate activation can result in pathological conditions including autoimmune diseases, cancer, and metabolic dysfunctions (12, 13). Thus, while the cGAS-STING pathway is vital for immune surveillance, it can also have detrimental consequences when improperly activated, indicating its dual role as both a protective and a potentially harmful signaling mechanism.

cGAS activity is intricately regulated through spatial and temporal mechanisms. Originally identified as a cytosolic DNA sensor, cGAS has also been localized in the nucleus, where it associates closely with chromatin. This nuclear localization appears to prevent cGAS from activating in response to self-DNA, as it is sequestered away from cytosolic compartments (14). Recent studies have shown that cGAS binds to the histone H2A-H2B heterodimer in nucleosomes, which inhibits its dimerization and enzymatic activation (15, 16). Additionally, cGAS has been found on the plasma membrane of macrophages, where its localization may also prevent activation by self-DNA during genotoxic stress (17). Beyond compartmentalization, cGAS activity is influenced by various post-translational modifications, including phosphorylation, acetylation, ubiquitination, glutamylation, and SUMOylation, each modulating its function in response to cellular cues (18, 19). The regulation of cGAS by these modifications underscores the complexity of its role in cellular signaling and immune responses.

In summary, the cGAS-STING signaling pathway exemplifies a sophisticated mechanism of immune detection that integrates pathogen recognition with the regulation of inflammation. Its dual nature as a protector against infections and a potential contributor to disease states highlights the need for further exploration of its functions, particularly in the context of metabolic disorders and other pathophysiological conditions. Understanding the intricate regulation of this pathway could provide new therapeutic targets for managing diseases linked to dysregulated immune responses and metabolism.

## 3 Post-translational modification of cGAS

The cGAS-STING pathway is closely controlled to protect the host from excessive immune activation, which could be harmful. Overstimulation of IFN production due to unchecked cytosolic cGAS activity has been linked to autoimmune conditions (1). Thus, the precise regulation of cGAS-STING signaling is essential for ensuring a balanced immune defense. Positive regulatory mechanisms facilitate the swift production of IFNs and pro-inflammatory cytokines, while negative feedback systems are in place to avoid overactivation of the immune system. Post-translational modifications play a key role in modulating or fine-tuning the cGAS-STING pathway.

### 3.1 Phosphorylation

#### 3.1.1 Phosphorylation of cGAS

The cGAS-STING pathway, integral for antiviral immune responses, is tightly regulated to ensure a balanced immune reaction and to prevent harmful consequences associated with overactivation, such as autoimmune diseases (20). One of the primary mechanisms for controlling cGAS activity involves post-translational modifications, particularly phosphorylation. Phosphorylation are critical post-translational modifications involved in regulating various cellular processes, this dynamic process enables cells to fine-tune cGAS function in response to viral infections or other DNA-damaging events (21, 22).

Phosphorylation can serve as a negative regulator of cGAS activity. For example, the kinase AKT plays a key role by phosphorylating human cGAS at Ser305 (and at Ser291 in murine cGAS), functioning as an “on/off” switch. This modification occurs near the catalytic site of cGAS and significantly hampers its ability to synthesize cGAMP, an essential molecule for activating the STING pathway. This inhibition of cGAS by AKT phosphorylation reduces the production of IFN-1 and other antiviral cytokines, ultimately enhancing viral replication, as demonstrated in herpes simplex virus 1 (HSV-1) infections (23). Inhibition of AKT, on the other hand, restores cGAS activity, boosting the immune response against the virus.

Once mitosis concludes, the ability of cGAS to sense cytosolic DNA is restored through the action of protein phosphatases such as PP1 and PP2, which dephosphorylate the inhibitory sites, enabling cGAS to resume its immune surveillance function. This reversible modulation underscores the importance of phosphorylation as a means to control cGAS activity based on cellular context, ensuring that immune responses are both timely and appropriately restrained to avoid autoimmunity (24).

#### 3.1.2 Phosphorylation and dephosphorylation of STING

Phosphorylation and dephosphorylation are critical for the modulation of the stimulator of STING pathway. As described above, oligomerized STING recruits TBK1, which phosphorylates STING at Ser366 (Ser365 in mice), a key step in activating downstream signaling pathways, such as IRF3-mediated IFN-1 production (25). In addition, C-terminal src kinase (CSK) phosphorylates STING at tyrosine residues (Tyr240 and Tyr245) to promote its aggregation and immune response activation during viral infections, such as herpes simplex virus 1 (HSV-1) (26). Similarly, protein phosphatase 6 catalytic subunit (PPP6C) acts as a negative regulator by dephosphorylating STING at Ser366, and its depletion significantly enhances STING activation in response to double-stranded DNA (dsDNA) stimulation (27). This fine-tuning ensures proper immune responses, preventing hyperactivation and maintaining immune homeostasis. Furthermore, epidermal growth factor receptor (EGFR) and spleen tyrosine kinase (SYK) can phosphorylate STING at Tyr245 and Tyr240, respectively, facilitating STING trafficking to the Golgi apparatus, which is essential for the production of IFN-1 (28). Conversely, the dephosphorylation of these sites by protein tyrosine phosphatases, such as tyrosine-protein phosphatase non-receptor types 1 and 2 (PTPN1/2), leads to STING degradation via a proteasomal pathway, thereby dampening immune responses (29).

Overall, the balance between phosphorylation and dephosphorylation is crucial for modulating STING activity. Dysregulation of this balance may contribute to pathological conditions, where hyperactivation or suppression of immune responses could drive disease progression.

### 3.2 Ubiquitination of cGAS

Ubiquitin is a small and highly conserved protein composed of 76 amino acids, including seven lysine residues and a single methionine residue (M1) (30). Ubiquitination, a significant post-translational modification, involves a cascade of enzymes: E1 activating enzymes, E2 conjugating enzymes, and E3 ligases, which facilitate the attachment of ubiquitin chains to target proteins. The process begins with ubiquitin forming a thioester bond with the E1 enzyme, which is then transferred to E2. Subsequently, the E2-ubiquitin complex interacts with an E3 ligase, forming an isopeptide bond between the carboxyl group of Gly-76 in ubiquitin and the lysine residue of the substrate (31, 32). This modification is crucial for the regulation of various cellular processes, including the cGAS-STING signaling pathway, which is vital for controlling antiviral immune responses.

The first E3 ubiquitin ligase identified to mediate the ubiquitination of cGAS was ring finger protein 185 (RNF185), which contains a RING domain that interacts directly with cGAS. RNF185 specifically facilitates K27-linked ubiquitination, which enhances the enzymatic activity of cGAS (33). The deletion of RNF185 reduces the activation of IRF3 triggered by cytoplasmic DNA (34). Another E3 ligase, ring finger protein interacting with C kinase (RINCK), also modulates cGAS activity by inducing its monoubiquitination. RINCK plays a key role in the synthesis of cGAMP, and its absence leads to compromised defenses against DNA viruses (35). Furthermore, TNF receptor-associated factor 6 (TRAF6), an additional E3 ubiquitin ligase, promotes cGAS activation and downstream IFN-1 production through specific ubiquitination of cGAS (36). Another significant player in this process is tripartite motif-containing protein 56 (TRIM56), which facilitates the ubiquitination of cGAS at Lys335, promoting its dimerization, DNA binding, and cGAMP synthesis, all of which contribute to antiviral immune responses (37, 38).

In addition to these direct interactions, some DUBs regulate cGAS indirectly. For instance, upon HSV-1 infection, USP14 is recruited and works alongside TRIM14 to remove K48-linked ubiquitin from cGAS at Lys414 (39). This process prevents autophagic degradation of cGAS, stabilizing it and promoting the expression of IFN-1, which enhances the organism’s antiviral defense capabilities (40).

## 4 cGAS/STING signaling in innate immunity

The cGAS-STING signaling pathway serves as a vital mechanism for the host’s defense against pathogen invasion, playing a crucial role in immune responses. This pathway is activated by a range of substrates, including both pathogen-derived DNA from viruses and self-derived DNA, such as mtDNA and genomic DNA. cGAS effectively recognizes various DNA viruses, including Kaposi’s sarcoma-associated herpesvirus (KSHV), vaccinia virus (VACV), herpes simplex virus type-1 (HSV-1), and retroviruses such as human immunodeficiency virus (HIV) and West Nile virus (WNV) (41, 42). Additionally, cGAS is involved in the immune response to several bacterial pathogens, such as *Listeria monocytogenes* and *Staphylococcus aureus* (43).

When cGAS detects dsDNA, it becomes activated and produces the second messenger cGAMP. This molecule binds to STING, prompting its translocation from the endoplasmic reticulum (ER) to the Golgi apparatus, leading to the production of IFN-β and other pro-inflammatory mediators. This signaling cascade enhances the host’s ability to eliminate invading pathogens. Notably, certain viral infections can induce oxidative stress or promote the release of interleukin-1β (IL-1β), which results in mtDNA escaping into the cytoplasm, further amplifying IFN-1 response (44, 45). In bacterial infections, mt DNA can be generated in response to lipopolysaccharides (LPS) and can also activate STING through direct interaction with cyclic dinucleotides (CDNs). While the cGAS-STING pathway is critical for antimicrobial defense, its activation by self-DNA can also lead to autoimmune conditions such as systemic lupus erythematosus (SLE) and rheumatoid arthritis. A clinical study indicated that 15% of SLE patients exhibited elevated levels of cGAMP despite carrying wild-type alleles for the exonuclease treX1, suggesting a complex underlying genetic basis (46). Moreover, cGAS/STING activation linked to DNA damage has been implicated in neurodegenerative diseases, including Parkinson’s disease, where it contributes to chronic inflammation (47). Recent research has further illuminated the cGAS-STING pathway’s role in cancer biology, proposing mechanisms for its anti-tumor effects. These mechanisms include promoting cellular senescence and enhancing cytokine production, which collectively stimulate immune responses against tumors (48, 49). This suggests that targeted activation of the cGAS-STING pathway could serve as a promising strategy for cancer immunotherapy (50). However, inappropriate activation of this signaling pathway has also been associated with chronic diseases, such as macular degeneration and non-alcoholic steatohepatitis (51, 52).

Despite its beneficial roles, various pathogens have developed strategies to evade the host immune response by targeting cGAS or STING. This can occur through mechanisms such as the degradation of signaling proteins or disruption of their pathways. For example, during HSV-1 infection, the viral protein USP21 negatively regulates IFN-1 signaling via the deubiquitination of STING, while other viral proteins can inhibit STING’s function by preventing its dimerization or cleaving it (53, 54). Additionally, some viruses, like hepatitis B virus (HBV), utilize protective mechanisms to shield their DNA from detection.

Understanding these complex interactions and mechanisms will be essential for developing targeted therapeutic strategies aimed at modulating this pathway in various clinical contexts.

## 5 cGAS/STING signaling in cardiometabolic diseases

### 5.1 cGAS/STING signaling and cardiovascular diseases

#### 5.1.1 Epidemiology and risk factors of cardiovascular diseases

Cardiovascular diseases (CVDs) including atherosclerosis (AS), heart failure, myocardial infarction (MI), coronary artery disease, and aortic aneurysm and dissection (AAD), are among the most common and deadly health conditions globally. Their incidence is increasing, with cases appearing in younger populations due to a combination of genetic, environmental, and lifestyle factors. Research has shown that age and sex play significant roles in CVD risk, with men being more prone to these diseases, while women typically develop them 10–15 years later (55, 56). Notably, conditions such as aortic aneurysm, one of the deadliest surgical CVDs, see mortality rates climb by 1–2% per hour after onset, with about 50% of patients succumbing before reaching the hospital. AS, cardiac hypertrophy, and hypertension further contribute to CVDs by triggering inflammatory responses in endothelial cells, largely due to mitochondrial dysfunction, endoplasmic reticulum stress, unfolded proteins, and macrophage infiltration (57–59). These mechanisms often lead to tissue fibrosis in advanced stages, highlighting the need for a deeper understanding of the intrinsic factors driving CVD progression.

Mounting evidence underscores the role of both innate and adaptive immune systems in CVD development, with immune dysregulation acting as a critical factor. Damage to host tissue, whether from endogenous or exogenous DNA, can lead to immune homeostasis disruption and inflammatory responses, contributing to the onset of CVDs. Ectopic DNA, resulting from cell damage or infection, is particularly harmful, triggering inflammatory cascades (60). Normally, enzymes like Three-prime repair exonuclease 1 (TREX1) act to degrade cytosolic DNA, preventing its accumulation and subsequent activation of IFN-1 pathway, which would otherwise provoke inflammation (61). Studies have shown that TREX1 deficiency in mice leads to increased mortality due to IFN-1 pathway activation, and these mice develop inflammatory myocarditis, resulting in cardiomyopathy and heart failure (62).

#### 5.1.2 Immune dysregulation and the role of cGAS/STING in CVDs

The discovery of the cGAS-STING pathway has provided new insights into the immune mechanisms behind CVDs. This pathway serves as a crucial sensor for cytosolic DNA, activating immune responses to maintain homeostasis. LPS triggers caspase-11 activation, which in turn activates GSDMD, resulting in the release of mtDNA from damaged mitochondria. In mouse models lacking TREX1, the absence of cGAS leads to complete protection from lethality, drastically reducing IFN-1-driven inflammation and preventing the production of autoantibodies (63). Additionally, cGAS-deficient mice exhibit reduced cardiac inflammation, less ventricular dilatation, and improved heart function (64, 65). These findings suggest that cGAS-STING is a central player in controlling DNA-induced inflammation in the cardiovascular system, offering potential therapeutic targets for the treatment of CVDs. The cGAS-STING signaling pathway plays a significant role in driving AS, myocardial damage, and impaired AS, all of which contribute to inflammatory responses in endothelial cells and macrophages (66). For instance, IRF3 has been implicated in the development of AS in both humans and murine models (67). Increased IRF3 expression has been observed in the endothelial layer of atherosclerotic arteries and within macrophages of atheromatous plaques in patients with coronary artery disease and hyperlipidemic ApoE^−/−^ mice. Knocking out IRF3 in hyperlipidemic mice has been shown to significantly reduce AS progression by decreasing macrophage infiltration, lipid accumulation, and inflammation, while also reducing necrotic core formation (68). Additionally, IRF3 depletion in endothelial cells was found to be more effective in reducing AS than its depletion in bone marrow-derived cells (67).

**Figure 2:**
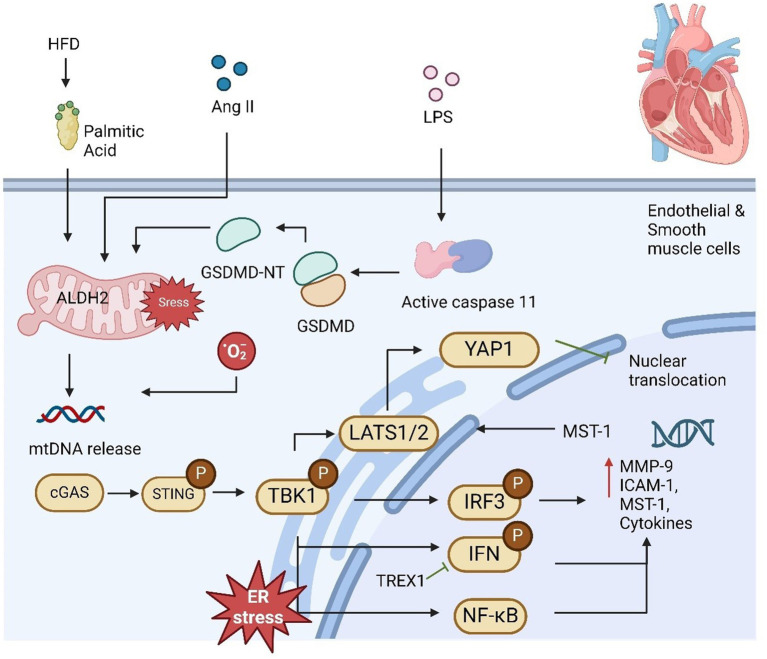
cGAS-STING pathway plays a significant role in mediating cardiovascular disease. High-fat diet (HFD) or palmitic acid (PA) exposure increases mitochondrial stress and lowers mitochondrial ALDH2 activity due to reduced melatonin levels, leading to impaired mitochondrial function and the release of mtDNA. Additionally, LPS activates caspase-11, which triggers GSDMD activation, further contributing to mtDNA release from damaged mitochondria. This mtDNA subsequently activates the STING-IRF3 pathway, which promotes macrophage infiltration and the development of cardiac fibrosis. DNA fragmentation in smooth muscle cells (SMCs) facilitates the transfer of DNA to macrophages, stimulating MMP-9 expression and contributing to extracellular matrix formation. Once activated, IRF3 translocates to the nucleus and induces MST1 expression, promoting YAP phosphorylation and suppressing cyclin D transcription, thereby inhibiting endothelial cell angiogenesis. Moreover, it upregulates ICAM-1 and VCAM-1 expression, enhancing inflammation and promoting SMC proliferation, contributing to proatherogenic effects.

AS is marked by lipid accumulation, immune cell infiltration, and foam cell formation in large arteries, leading to vessel narrowing, ischemia, and severe clinical outcomes. IFN-1 play a key role in atherogenesis by enhancing macrophage activity and immune cell responses that worsen AS (69). The cGAS-STING pathway has emerged as a critical sensor of aberrant DNA, activating IFN-1 and promoting inflammation, VSMC phenotypic switching, and plaque instability. Studies show that cGAS or STING deficiency reduces plaque vulnerability by preventing premature VSMC senescence and preserving fibrous caps, positioning the pathway as a therapeutic target (70). In addition, mitochondrial DNA release due to mitochondrial damage activates cGAS-STING in endothelial cells, increasing monocyte adhesion and exacerbating AS (71). Bacterial LPS further induces mtDNA release via gasdermin D cleavage, enhancing cGAS-STING signaling (Figure 2). The pathway also drives inflammation in macrophages through TBK1 and NF-κB activation (72–74). While other DNA sensors may be involved, the full connection between DNA damage and AS progression remains unclear. In summary, the cGAS-STING pathway significantly contributes to AS development and plaque rupture through its regulation of inflammation and vascular cell behavior. Targeting this pathway offers potential for new therapies aimed at reducing plaque instability and treating AS.

The development of aortic aneurysm and dissection (AAD) is primarily driven by the progressive loss of smooth muscle cells (SMCs) and degradation of the extracellular matrix (ECM), eventually leading to aortic dilation, dissection, and rupture (75–77). Despite extensive research, the exact molecular mechanisms underlying these processes remain under investigation. Recent studies have identified the cGAS-STING signaling pathway as a critical factor in the pathogenesis of AAD, providing new insights into its development (66, 78). In the setting of AAD, increased cytoplasmic DNA release from damaged SMCs and macrophages has been shown to activate STING. Specifically, DNA from SMCs in sporadic AAD induced by high-fat diet and angiotensin II (Ang II) in mouse models is phagocytosed by macrophages, initiating the activation of STING and its downstream signaling via IRF3. This cascade leads to the upregulation of matrix metalloproteinase-9 (MMP-9), which directly promotes ECM degradation, contributing to aortic thickening, dissection, and rupture (66). This pathological process is significantly attenuated in STING-deficient mice, where aortic dilation and rupture are markedly reduced. Furthermore, reactive oxygen species (ROS)-induced DNA damage results in the continuous release of DNA fragments, which further stimulate the cGAS-STING-TBK1-IRF3 axis, exacerbating SMC death (79). In a single-cell analysis, activation of STING-IRF3 signaling was shown to drive a shift in SMCs from their normal contractile state to a pro-inflammatory phenotype (80). This phenotypic shift, characterized by chromatin remodeling, is a key contributor to AAD progression. In contrast, STING knockout mice exhibited resistance to this stress-induced inflammatory transformation, maintaining the integrity of the SMC population (80).

#### 5.1.3 Vascular remodeling and STING-dependent signaling

STING has been found to play a critical role in promoting SMC apoptosis and necrosis, which is mediated through IFN-1 and TNF receptor signaling pathways (81). The release of damaged SMC DNA into macrophages triggers STING-dependent IRF3-MMP-9 signaling, further degrading the ECM and driving the degenerative changes associated with AAD (66). TBK1-IRF3 signaling also exacerbates fructose-induced cardiac injury, and suppression of this pathway through the NLRP4 inflammasome dampens inflammation and hypertrophy. In mouse models of aortic banding-induced cardiac hypertrophy, STING knockdown alleviates cardiac remodeling and inhibits fibrosis and inflammatory responses (82). Additionally, STING-mediated ER stress has been implicated in pathological hypertrophy. STING activation in macrophages during myocardial infarction (MI) promotes inflammation, fibrosis, and apoptosis in both cardiomyocytes and cardiac fibroblasts (82). In the context of obesity-induced cardiac dysfunction, overactivation of the cGAS-STING pathway leads to increased inflammation and impaired mitochondrial autophagy. Furthermore, in models of Alzheimer’s disease (AD)-related cardiac dysfunction, melatonin treatment alleviated myocardial remodeling and contractile dysfunction through restoration of mitochondrial integrity via the cGAS-STING-TBK1 pathway (83). Interestingly, contrary to traditional views, excessive mtDNA leakage was shown to inhibit, rather than activate, the cGAS-STING pathway by depleting intracellular cGAS, suggesting that effective STING activation may be contingent on maintaining mitochondrial integrity and sufficient intracellular cGAS levels (84). Further research is needed to elucidate this phenomenon.

#### 5.1.4 cGAS/STING signaling and metabolic regulation in CVDs

Emerging evidence suggests a link between the cGAS-STING pathway and metabolic regulation in CVDs. Research has demonstrated that cGAS-STING activation influences the expression of key regulators of lipid metabolism, including sterol regulatory element-binding proteins (SREBPs) and fatty acid-binding proteins (FABPs) (85). For example, in atherosclerotic lesions, cGAS-STING activation promotes the release of pro-inflammatory cytokines, enhancing macrophage foam cell formation by disrupting cholesterol efflux pathways (1). Moreover, mitochondrial stress-induced mtDNA release activates cGAS-STING in vascular endothelial cells, exacerbating lipid peroxidation and monocyte adhesion (86). Additionally, there is growing interest in the cGAS-STING pathway’s role in regulating mitochondrial function and its impact on oxidative stress and lipid metabolism. The inhibition of this pathway has been shown to reduce lipid accumulation in preclinical models, suggesting its potential as a therapeutic target for managing lipid dysregulation in CVDs (87, 88).

These findings highlight the dual role of cGAS-STING signaling in mediating inflammatory responses and lipid metabolic processes, underscoring its significance in the metabolic regulation of cardiovascular diseases. Further studies are warranted to explore its therapeutic potential in targeting metabolic pathways for improved cardiovascular outcomes.

### 5.2 cGAS/STING signaling in metabolism

#### 5.2.1 cGAS/STING signaling and obesity

The canonical STING-IRF3 signaling pathway plays a significant role in driving inflammation in adipose tissue under conditions of obesity (89, 90). Activation of STING in adipocytes has been shown to enhance the phosphorylation of Jun N-terminal kinase (JNK) and NF-κB in response to LPS stimulation, further amplifying proinflammatory responses in adipocytes and hepatocytes. In addition, the deficiency of disulfide bond A oxidoreductase-like protein (DsbA-L) in adipose tissue during obesity leads to mitochondrial dysfunction. This dysfunction triggers the release of mtDNA, which activates the cGAS-STING pathway, leading to a cascade that decreases cyclic AMP (cAMP) levels and inhibits protein kinase A (PKA) signaling. These disruptions contribute to obesity-induced inflammation and metabolic imbalances. Moreover, the STING-IRF3 axis regulates intracellular adhesion molecule-1 (ICAM-1) expression in endothelial cells, promoting macrophage infiltration and high-fat diet (HFD)-induced endothelial inflammation within adipose tissue. Collectively, overnutrition, cytokines, and cytosolic DNA serve as activators of cGAS-STING signaling in various adipose cell types, contributing to widespread inflammation and metabolic dysfunction.

Beyond inflammation, the STING-IRF3 pathway has been implicated in the dysregulation of insulin signaling, which is a key feature of obesity-related insulin resistance (91). Insulin signaling typically involves components like the insulin receptor, IRS1, PI3K, AKT, and GLUT4, with phosphorylation events driving the translocation of GLUT4 and intracellular glucose transport. However, in the context of obesity, interactions between insulin signaling and inflammatory pathways, including STING-IRF3, contribute to insulin resistance (92, 93). Studies have shown that IRF3 deficiency reduces phosphorylation of AKT and IRS1 at Tyr608 in the livers of HFD-fed mice, impairing insulin signaling (94). Conversely, in white adipose tissue, IRF3 knockout increases GLUT4 expression and enhances AKT phosphorylation, thereby maintaining glucose homeostasis (95).

Studies have demonstrated that IRF3, part of the cGAS-STING pathway, plays a protective role against diet-induced hepatic insulin resistance. By inhibiting the proinflammatory IKKβ/NF-κB pathway, IRF3 mitigates insulin resistance in the liver. Mice lacking IRF3 showed reduced insulin receptor substrate 1 (IRS1) and protein kinase B (AKT) phosphorylation in the liver, worsening insulin resistance (96). However, in 3T3-L1 adipocytes, IRF3 contributed to insulin resistance induced by TLR ligands, while IRF3 knockout improved glucose homeostasis by enhancing glucose uptake and increasing GLUT4 expression and insulin signaling in adipose tissue (97). These findings suggest that the role of IRF3 in insulin resistance is tissue-specific, necessitating further research with tissue-specific IRF3 knockouts.

TBK1, a downstream effector of STING, also interacts with insulin signaling pathways. In obese Zucker rats and HFD-fed mice, TBK1 phosphorylates the insulin receptor on Ser994, contributing to insulin resistance (98). Moreover, inhibiting TBK1 enhances insulin-stimulated AKT phosphorylation, improving insulin sensitivity. TBK1 has further been linked to reduced expression of the insulin receptor and AKT in the hippocampus of obese mice (99, 100). These findings suggest that the cGAS-STING-TBK1-IRF3 axis plays a crucial role in the crosstalk between inflammation and insulin resistance, although further studies are needed to clarify the exact mechanisms underlying these interactions.

#### 5.2.2 cGAS/STING signaling and thermogenesis during obesity

**Figure 3:**
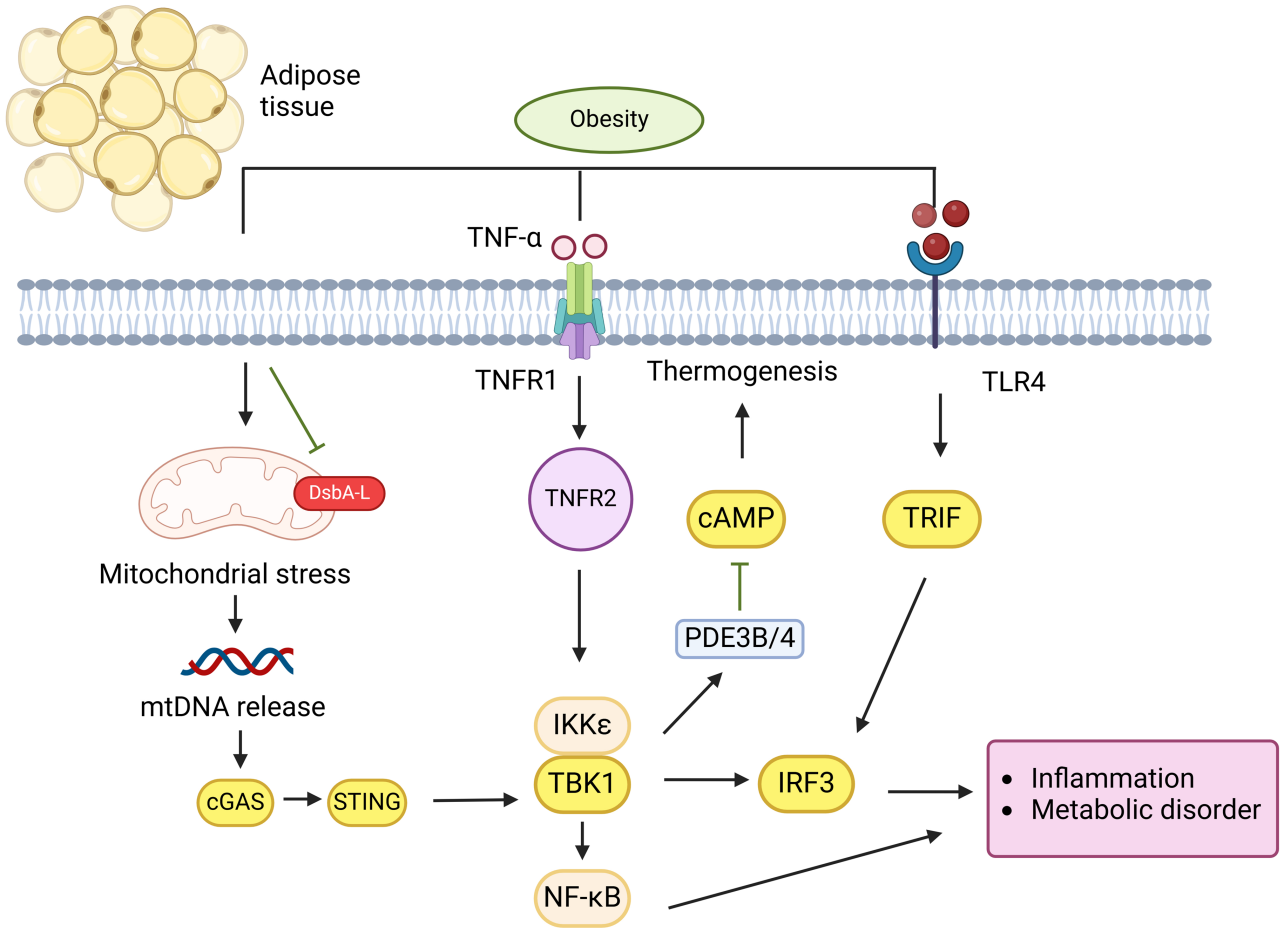
cGAS-STING pathway plays a critical role in mediating obesity in adipose tissue. Overnutrition induces mitochondrial stress and dysfunction, leading to the release of mtDNA in adipocytes due to DsbA-L inhibition. This mtDNA is detected by cGAS, which subsequently activates the STING-TBK1-IRF3 pathway, resulting in IFN-1 production or activation of the TBK1-NF-κB pathway, both of which promote the release of proinflammatory cytokines. Similarly, LPS activate IRF3 via TLR4 receptor binding. The activation of TNFR1 by TNF-α and TLR4 by palmitate synergistically increases CCL2/MCP-1 production, promoting macrophage infiltration and inflammation in obesity. Additionally, IRF3 suppresses GLUT4 expression in adipocytes, leading to insulin resistance. On the other hand, STING activation of TBK1/IKKε inhibits PDE3B/4 expression, which suppresses cAMP-PKA signaling, thereby reducing thermogenesis and contributing to obesity attenuation.

In addition to its role in regulating insulin sensitivity and action, the activation of the cGAS-STING pathway has been linked to the regulation of energy balance (4). Obesity arises from excessive energy intake and/or inadequate energy expenditure, partly due to impaired thermogenic activity in brown or beige fat. These fat tissues, which usually dissipate energy as heat via thermogenesis due to their high mitochondrial density, are compromised. Mice with fat-specific DsbA-L knockout exhibited increased mitochondrial stress, greater activation of the cGAS-STING pathway in adipose tissue, and reduced expression of thermogenic genes when exposed to cold. These mice also showed decreased energy expenditure and gained more weight. Conversely, STING inactivation or reduced mitochondrial stress through fat-specific DsbA-L overexpression protected mice against obesity induced by a high-fat diet. In DsbA-L-deficient adipocytes, suppression of cGAS or STING through shRNA or inhibition of the STING downstream kinase TBK1/IKKe led to increased phosphorylation of protein kinase A (PKA) and enhanced expression of the uncoupling protein 1 gene, indicating a cell-autonomous effect of the cGAS-STING pathway on thermogenic gene regulation. Treatment of primary adipocytes with 2′3′-cGAMP, which activates downstream STING signaling, increased the activity of phosphodiesterases PDE3B/PDE4 and lowered intracellular cAMP levels. In contrast, STING knockout significantly elevated both basal and β3-adrenoceptor agonist CL316243-induced cAMP levels in primary adipocytes. These findings suggest that mitochondrial stress-induced activation of the cGAS-STING pathway acts as a suppressor of thermogenesis in adipose tissue by inhibiting cAMP-PKA signaling and reducing thermogenic gene expression. Supporting this, adipose-specific knockout of TBK1 reduced HFD-induced obesity by increasing energy expenditure, likely due to enhanced AMPK activity, which promotes catabolic processes (101). Additionally, treating obese mice with amlexanox, an inhibitor of TBK1 and IKKe, raised energy expenditure by boosting thermogenesis (102). The TBK1 target IRF3 has been identified as a strong repressor of thermogenic gene expression and oxygen consumption in adipocytes, and mice lacking IRF3 displayed increased beige fat development and energy expenditure (103) (Figure 3). Furthermore, abnormal activation of type I IFN signaling in brown adipocytes caused significant mitochondrial dysfunction and reduced thermogenic capacity. These data reveal that adipose cGAS-STING-IFN signaling plays a negative role in the regulation of thermogenesis and energy expenditure. However, further research using adipose tissue-specific knockout models for cGAS and STING is needed to confirm their roles in energy homeostasis.

#### 5.2.3 cGAS/STING signaling and lipid metabolism

Cholesterol and fatty acid synthesis are tightly regulated by SREBP1 and SREBP2, which are key transcription factors belonging to the basic helix-loop-helix leucine zipper family (104, 105). These SREBPs are produced as precursors in the endoplasmic reticulum (ER) and processed in the Golgi apparatus into their active forms, where they become functional transcription factors. The transport of SREBPs from the ER to the Golgi requires their interaction with SREBP cleavage-activating protein (SCAP), which facilitates their movement via COPII-coated vesicles (9).

Studies reveal that lipid metabolism and IFN response are co-regulated, allowing innate immune cells to modulate metabolism during viral infections (106, 107). For instance, treating bone marrow-derived macrophages (BMDMs) with viral or IFN-stimulating agents decreased the synthesis of cholesterol and long-chain fatty acids while increasing their cellular uptake through an IFNAR-dependent pathway, demonstrating the disruption of lipid metabolism during innate immune activation (108). Interestingly, the relationship between innate immune signaling and cholesterol metabolism is bidirectional, as silencing SCAP or SREBP2, which shifts metabolism from cholesterol synthesis to import, activated the cGAS-STING pathway and enhanced type I IFN signaling, increasing viral resistance (109). Conversely, cholesterol supplementation reduced IFN gene expression, counteracting the antiviral phenotype.

Research has also shown that SREBP2 trafficking by SCAP is essential for proper STING translocation and downstream signaling (110). SCAP interacts with STING, acting as a scaffold to recruit IRF3 to the perinuclear region or Golgi microsomes, triggering antiviral signaling (107). Inhibition of SCAP impairs STING-mediated IRF3 signaling, increasing susceptibility to infections like HSV-1. More recently, Chu et al. (110) identified Niemann–Pick type C1 (NPC1), a lysosomal membrane protein, as a cofactor in STING’s lysosomal degradation. NPC1 knockout disrupted cholesterol export, facilitating STING activation by its retention in the SCAP-SREBP2 complex and leading to increased STING signaling. In addition to cholesterol metabolism, disturbances in fatty acid metabolism also activate the cGAS-STING pathway. Inhibition of FABP5, a lipid chaperone involved in fatty acid uptake and transport, activated cGAS-STING-mediated type I IFN signaling (111). Disruptions in FABP5 reduced fatty acid elongation and desaturation, impaired mitochondrial function, and triggered mtDNA release, which activated the cGAS-STING pathway in regulatory T cells. Similar findings by Yuan et al. (111) showed that palmitic acid-induced mtDNA release activated cGAS-STING signaling, suggesting that fatty acid supplementation enhances antiviral defenses.

These findings indicate a strong link between the cGAS-STING pathway, fatty acid metabolism, and type I IFN signaling. However, further research is required to understand whether this reciprocal regulation also occurs in metabolic tissues like the liver, which plays a central role in cholesterol and fatty acid metabolism, and how the innate immune system contributes to lipid homeostasis in these tissues.

#### 5.2.4 cGAS/STING signaling and glycerolipid metabolism

The cGAS-STING signaling pathway plays a crucial role in the pathogenesis of glycerolipid metabolism, which includes both nonalcoholic and alcoholic fatty liver diseases. Nonalcoholic fatty liver (NAFL), characterized by excess fat accumulation in the liver of individuals who consume little to no alcohol, can progress into nonalcoholic steatohepatitis (NASH), a more severe inflammatory condition that increases the risk of cirrhosis, hepatocellular carcinoma, and liver failure, contributing to heightened mortality in obese patients (112, 113). Elevated levels of cGAS-STING signaling components, including cGAS, STING, and IRF3, have been observed in both human NAFL and NASH patients, as well as in high-fat diet (HFD) or NASH mouse models (9, 114). Mice deficient in STING exhibit reduced hepatic steatosis, fibrosis, and inflammation when subjected to either an HFD or a methionine- and choline-deficient (MCD) diet, suggesting the pathway’s involvement in diet-induced NAFLD/NASH (51). Furthermore, silencing STING or IRF3 in human hepatic cells significantly reduces fatty acid-induced hepatic inflammation and apoptosis by inhibiting the NF-κB pathway and lowering inflammatory cytokine levels (115).

However, the specific role and expression of STING in the liver remain ambiguous. While some evidence suggests that STING is expressed predominantly in nonparenchymal cells such as macrophages—including monocyte-derived macrophages (CCR2^+^, S100A9^+^), Kupffer cells (CD68^+^), and CD163^+^ macrophages, other studies point to its expression in hepatocytes, where it contributes to hepatocyte injury and apoptosis (116, 117). Interestingly, transplantation of bone marrow from STING-deficient mice into wild-type mice led to a reduction in HFD-induced hepatic steatosis, while the reverse scenario restored hepatic fat accumulation and inflammation, highlighting the critical role of macrophage STING in diet-induced liver dysfunction. Despite these findings, further research is needed to clarify the tissue-specific roles of cGAS and STING in NAFLD/NASH.

The role of IRF3 in NAFLD development has been a subject of controversy, as its functions vary depending on the signaling pathway involved. Some research suggests that cytoplasmic IRF3 interacts with IKKβ, inhibiting NF-κB activation, and that reduced IRF3 levels may protect hepatocytes from HFD-induced hepatic steatosis, inflammation, and macrophage infiltration (96). Conversely, another study demonstrated that IRF3 inhibits lipogenesis by binding directly to the Scd1 promoter, which encodes a rate-limiting enzyme for fat synthesis (118). While TRIF-IRF3 signaling appears to inhibit lipogenesis, STING-IRF3 signaling has been shown to promote lipid accumulation, inflammation, and apoptosis in hepatocytes (115). In obese mice and human hepatocytes, elevated STING and IRF3 levels have been linked to increased proinflammatory signaling and markers of apoptosis, with silencing either molecule leading to reduced inflammation, increased glycogen storage, and diminished fat deposition (115).

Several studies have highlighted the significant role of the STING-IRF3 pathway in promoting NAFLD/NASH through enhanced lipid accumulation and inflammatory responses. Elevated TBK1 phosphorylation, a downstream target of STING, has been observed in HFD-fed mice, and inhibiting TBK1 via amlexanox or gene knockout significantly reduced hepatic steatosis, inflammation, and macrophage infiltration (102, 119). Cho et al. (120) demonstrated that the cGAS-STING-TBK1 axis mediates p62 phosphorylation, which leads to protein inclusions, a key marker of NASH, in hepatocytes exposed to saturated fatty acids and in HFD-fed obese mice. Additionally, STING and TLR9 synergistically promote mitochondrial DNA (mtDNA)-induced inflammatory responses in hepatocytes, especially through Kupffer cell activation (51). The involvement of STING in glucose regulation via IRF3, independent of body weight effects, further underscores its complex role in liver metabolic function.

The cGAS-STING pathway also plays a pivotal role in liver fibrosis, a key complication of NASH. Mice lacking STING show reduced expression of fibrosis markers such as αSMA, TGF-β1, and Collagen 1A1 following an MCD diet, while STING knockout attenuates chronic inflammation-induced liver fibrosis (9). STING activation in hepatic stellate cells (HSCs) has been linked to increased TGF-β1-induced fibrosis, suggesting a mechanism where macrophage STING triggers fibrosis through paracrine signaling to HSCs. Additionally, the STING-IRF3 axis has been implicated in connecting mitochondrial stress-induced hepatocyte death to liver fibrosis, linking inflammation to secondary injury (117).

**Figure 4:**
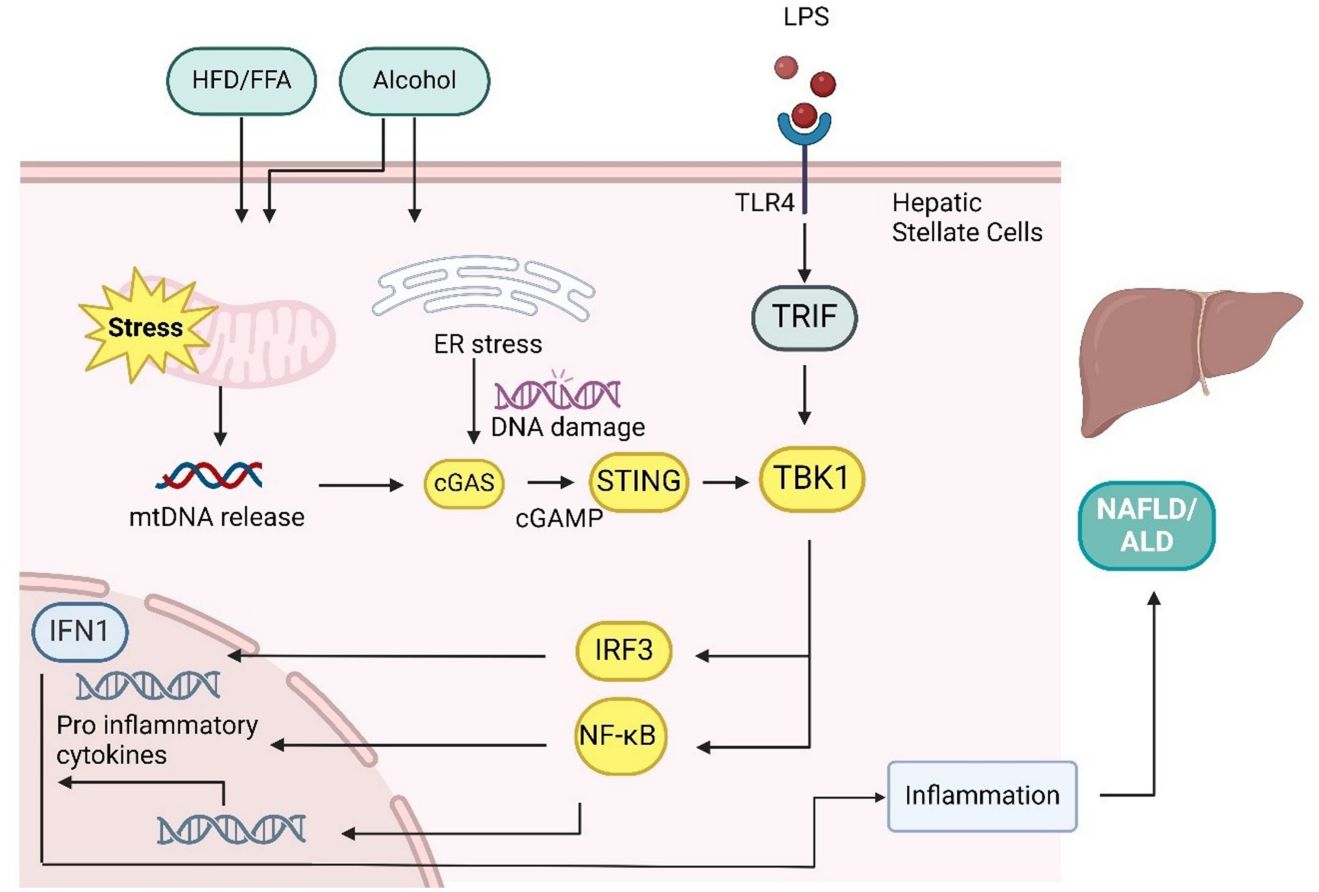
cGAS-STING pathway in the liver plays a key role in mediating NAFLD/NASH/ALD. Mitochondrial stress caused by methionine-choline-deficient (MCD) diets, high-fat diets (HFD), free fatty acids (FFAs), and LPS leads to the release of mtDNA. In hepatocytes and hepatic stellate cells, mtDNA activation triggers inflammatory responses similar to those in adipocytes, resulting in elevated inflammation, lipid accumulation, and liver fibrosis through proinflammatory cytokine production. The transport of cGAMP from hepatocytes to immune cells via connexin 32 (CX32) spreads these inflammatory effects. In addition, alcohol-induced ER stress and LPS/TLR4 signaling can activate the cGAS-STING-IRF3 pathway, leading to apoptosis. However, IRF3 also suppresses Scd1 transcription through TLR3/TRIF signaling, which inhibits adipogenesis.

In alcohol-related liver disease (ALD), the cGAS-STING pathway is similarly important. Alcohol-induced hepatotoxicity and oxidative stress contribute to a range of liver conditions, from steatosis to cirrhosis and liver cancer, but no effective treatment currently exists (94). In ALD, IRF3 is activated in response to ER stress in hepatocytes, where it works with the apoptotic protein Bax to initiate STING-mediated apoptosis (121). Alcohol consumption releases mtDNA in hepatocytes, which activates cGAMP. Connexin 32 (Cx32) facilitates the intercellular transfer of cGAMP, spreading inflammation signals from hepatic parenchymal to nonparenchymal cells, amplifying the secondary inflammatory response in alcoholic fatty liver (122). In addition, cGAS and IRF3 knockout models show protection against ALD, underscoring the pathway’s therapeutic potential. The main signaling pathway is as in Figure 4.

Overall, the cGAS-STING pathway is critical in nutrient stress-induced NAFLD, NASH, ALD, and even hepatocellular carcinoma (97). Its involvement in both hepatocytes and macrophages makes it a promising therapeutic target for treating a wide range of liver diseases.

## 6 Pre-clinical development of cGAS-STING pathway inhibitors

The cGAS-STING signaling pathway has emerged as a key target for therapeutic intervention due to its role in chronic inflammation, autoimmunity, and metabolic disorders. Various pre-clinical studies have focused on developing inhibitors that can selectively modulate this pathway, aiming to reduce adverse inflammatory responses while preserving essential immune functions. This section summarizes several promising cGAS-STING inhibitors and their potential therapeutic applications.

### 6.1 Small-molecule inhibitors of STING

Small molecules, such as Vent-03, H-151, C-176, and C-178, inhibit STING by binding to its transmembrane domain, blocking its translocation to the Golgi apparatus, a crucial step for downstream signaling (123). In murine models of autoimmune diseases, H-151 has been shown to decrease IFN-1 production, which alleviates symptoms in models of systemic lupus erythematosus (SLE) and psoriasis (124). Notably, C-176 has demonstrated efficacy in reducing inflammatory markers in SLE models, highlighting its potential for treating autoimmune diseases linked to STING overactivation (125). Vent-03 has shown potential in pre-clinical studies, and it showed safety priority in well-tolerated doses far exceeding those planned for use in Phase 2 trials. Its efficacy in animal models highlights the potential for using cGAS-STING pathway inhibitors to manage inflammatory and metabolic conditions where the pathway’s chronic activation exacerbates disease progression (126).

### 6.2 Antagonists targeting cGAS/STING signaling

Direct inhibition of cGAS has been explored with compounds like RU.521 and G150. RU.521, for instance, directly binds to the cGAS enzyme, inhibiting its ability to synthesize cGAMP (127). Pre-clinical studies have shown that RU.521 is effective in preventing inflammatory responses in murine models of lupus and arthritis (127). Another compound, G150, inhibits cGAS by blocking its DNA-binding site, effectively reducing cGAMP production (128). Such cGAS-targeted approaches may provide a safer therapeutic option, especially in diseases where upstream inhibition of cGAS is preferred to reduce downstream immune activation. Moreover, there is increasing interest in cyclic dinucleotide (CDN) analogs and STING competitive inhibitors. For example, DMXAA and CRID3 are CDN analogs that act as competitive inhibitors, preventing the binding of natural CDNs to STING (129). Initially developed as anti-tumor agents, these compounds have shown promise in reducing chronic inflammation in pre-clinical models of cardiovascular and metabolic diseases. In AS models, DMXAA reduced vascular inflammation by downregulating pro-inflammatory cytokines and decreasing plaque formation, suggesting its utility in conditions driven by vascular inflammation (130, 131). In recognition of the interconnected nature of inflammatory pathways, dual inhibitors targeting cGAS-STING and other immune pathways are also being developed. Amlexanox, a dual inhibitor of TBK1 and IKKε, downstream kinases in the STING pathway, has shown potential in animal models of obesity and type 2 diabetes. It works by reducing STING-mediated inflammation, which in turn improves insulin sensitivity and metabolic health. Amlexanox-treated mice exhibit reduced adipose inflammation and improved glucose tolerance, making it a promising candidate for metabolic diseases associated with chronic inflammation (132). Peptide inhibitors, such as SNX9-derived peptides, are being investigated for their ability to inhibit cGAS dimerization, an essential step for its activation. These peptides have shown promise in *in vitro* studies by blocking cGAS activation in response to cytosolic DNA (133). Though still in early pre-clinical stages, peptide inhibitors may offer a more targeted approach with fewer off-target effects, holding potential for diseases where selective inhibition of the cGAS-STING axis is critical.

### 6.3 Therapeutic potential in cancer

While many inhibitors are aimed at reducing inflammation, cGAS-STING pathway activation has also shown potential in cancer immunotherapy, where stimulating immune responses against tumors is beneficial. Paradoxically, in the cancer context, STING agonists (such as ADU-S100) are in development to boost immune surveillance (134, 135). However, antagonists like H-151 and C-176 are also being investigated in cancers associated with chronic inflammation, where reducing STING activity could help minimize tumor-promoting inflammation, especially in hepatocellular carcinoma and pancreatic cancer models (136, 137).

While these pre-clinical studies have demonstrated the therapeutic promise of cGAS-STING inhibitors, clinical translation faces several challenges, including achieving tissue specificity, minimizing off-target effects, and ensuring adequate bioavailability. Ongoing research is focused on optimizing these inhibitors to enhance their safety profiles and efficacy. Nanoparticle-based delivery systems and tissue-specific targeting approaches are under exploration to improve the selective inhibition of cGAS-STING signaling in target tissues, potentially paving the way for precision therapies in inflammation-driven diseases.

## 7 Conclusions and prospectives

cGAS-STING signaling pathway, long recognized for its vital role in the detection of cytosolic DNA and initiation of innate immune responses, has been increasingly implicated in the regulation of metabolic processes. While initially appreciated for its antiviral and antitumor functions, recent evidence reveals that this pathway also plays a significant role in the modulation of lipid metabolism, particularly glycerolipid metabolism. This dual functionality positions the cGAS-STING axis at the intersection of immune regulation and metabolic control, adding complexity to its biological significance. Dysregulation of the pathway has been strongly linked to a number of metabolic disorders, including obesity, insulin resistance, and liver disease, as well as broader inflammatory conditions. As such, the pathway offers considerable potential as a therapeutic target for addressing metabolic dysfunctions, but its precise regulation in various tissues remains a subject of ongoing investigation.

The cGAS-STING pathway exerts its metabolic influence through mechanisms that involve not only immune cells but also adipocytes, hepatocytes, and other metabolic cells. For example, mitochondrial stress in adipocytes caused by overnutrition or fatty acid accumulation can lead to the release of mtDNA, which activates the cGAS-STING pathway. In turn, this activation promotes inflammatory responses that can exacerbate conditions like obesity and insulin resistance. Similarly, in hepatocytes, cGAS-STING signaling drives inflammation, lipid accumulation, and fibrosis, contributing to the progression of NAFLD and NASH. These observations highlight the diverse roles of this pathway across metabolic tissues, suggesting that its activation can induce a feedback loop that perpetuates metabolic dysfunction through chronic inflammation. One of the most compelling aspects of the cGAS-STING pathway is its ability to interact with multiple other signaling cascades that govern both immune and metabolic processes. For instance, components of this pathway can modulate key metabolic regulators such as the TLR4-NF-κB, JNK, AMPK, mTOR, and insulin signaling pathways. These interactions allow the cGAS-STING axis to play a central role in balancing immune responses with cellular metabolism. However, while transient activation of this pathway can provide necessary immune protection, prolonged or chronic activation is associated with detrimental effects. Sustained activation of the cGAS-STING pathway has been shown to contribute to persistent inflammation, leading to increased insulin resistance, lipid accumulation, and even tumorigenesis in certain contexts. Therefore, achieving precise control over the timing and magnitude of this pathway’s activation is critical for maintaining metabolic health and preventing disease progression.

Despite the advances in understanding the metabolic functions of the cGAS-STING pathway, several challenges remain in fully elucidating its mechanisms. One key challenge lies in determining how this pathway functions in a tissue-specific manner. Studies have shown that the effects of cGAS-STING activation can vary widely depending on the cell type involved. For instance, in adipocytes, STING activation has been linked to reduced thermogenesis and increased fat storage, whereas in hepatocytes, the same pathway contributes to inflammation and fibrosis. Additionally, the cGAS-STING pathway’s interaction with immune cells such as macrophages further complicates its role, as it can lead to immune cell infiltration into metabolic tissues, exacerbating inflammation and metabolic imbalance. Understanding these tissue-specific effects is crucial for developing targeted therapies that can modulate this pathway without inducing harmful side effects. Therapeutically, targeting the cGAS-STING pathway presents both opportunities and challenges. On one hand, the pathway’s central role in linking immune and metabolic responses makes it an attractive target for treating chronic inflammatory metabolic diseases. Inhibiting cGAS or STING has the potential to mitigate inflammation and improve metabolic outcomes in diseases such as obesity, insulin resistance, and NAFLD. Moreover, because cGAS-STING activation can lead to tumorigenesis in certain contexts, selectively inhibiting this pathway could also offer benefits in cancer prevention. On the other hand, selective modulation of this pathway is not without risks. Given its critical role in antiviral and antitumor immunity, indiscriminate inhibition of cGAS-STING could compromise the body’s ability to mount effective immune responses. Therefore, it is essential to develop therapeutic strategies that can precisely target the pathway in specific tissues or conditions, while preserving its protective immune functions. Future research will need to focus on addressing several key questions. First, it remains unclear how much of the observed metabolic dysfunctions linked to cGAS-STING are directly due to pathway activation within metabolic cells, as opposed to secondary effects resulting from immune cell infiltration and inflammation. Additionally, there is a need to explore how distinct regulatory mechanisms within different tissues influence the pathway’s function. The development of more sophisticated pharmacological agents, capable of specifically targeting cGAS-STING components in a tissue- and cell-specific manner, will be crucial for unlocking the therapeutic potential of this pathway.

In light of the tissue-specific challenges highlighted above, various experimental approaches can be implemented to investigate the role of cGAS-STING signaling across distinct tissue types. Advanced genetic tools, such as tissue-specific knockouts or conditional knockout models, can provide insights into the functional contributions of cGAS and STING within particular tissues, such as adipose, liver, and cardiovascular tissues. These models allow for the precise inactivation of cGAS-STING components in target organs, thereby isolating their tissue-specific effects on metabolism and inflammation. Furthermore, single-cell RNA sequencing can be employed to reveal cell-specific gene expression changes and identify distinct cell populations responding to cGAS-STING activation within heterogeneous tissues. This approach, combined with spatial transcriptomics, could help elucidate the microenvironmental context of cGAS-STING signaling, particularly in inflammatory or fibrotic settings. Lastly, imaging techniques like fluorescence microscopy using tagged versions of cGAS or STING, coupled with live-cell imaging, offer a real-time perspective on how these proteins localize and respond to stimuli within various cellular compartments across different tissues. Such imaging can help track protein translocation and activation dynamics, shedding light on tissue-specific regulatory mechanisms. These methodologies collectively offer pathways for overcoming the existing knowledge gaps and enhancing our understanding of the nuanced roles of cGAS-STING signaling in tissue-specific contexts.

In conclusion, the cGAS-STING signaling pathway represents a critical nexus between immune regulation and metabolic control, with implications for a wide range of diseases. While much progress has been made in understanding its role in innate immunity, its emerging functions in metabolism are only beginning to be fully appreciated. Continued research into the tissue-specific and cell-specific roles of this pathway will provide new insights into the mechanisms underlying metabolic disorders and may pave the way for novel therapeutic interventions aimed at restoring metabolic balance and enhancing health outcomes.

## Footnotes

^1^ https://www.proteinatlas.org/ENSG00000164430-CGAS/tissue

^2^ https://www.proteinatlas.org/ENSG00000184584-STING1/tissue

## References

[1] Decout A Katz JD Venkatraman S Ablasser A. The cGAS-STING pathway as a therapeutic target in inflammatory diseases. Nat Rev Immunol. (2021) 21:548–69. doi: 10.1038/s41577-021-00524-z, PMID: 33833439 PMC8029610

[2] Hopfner K-P Hornung V. Molecular mechanisms and cellular functions of cGAS-STING signalling. Nat Rev Mol Cell Biol. (2020) 21:501–21. doi: 10.1038/s41580-020-0244-x 32424334

[3] Bai J Cervantes C Liu J He S Zhou H Zhang B. DsbA-L prevents obesity-induced inflammation and insulin resistance by suppressing the mtDNA release-activated cGAS-cGAMP-STING pathway. Proc Natl Acad Sci USA. (2017) 114:12196–201. doi: 10.1073/pnas.1708744114 29087318 PMC5699051

[4] Bai J Cervantes C He S He J Plasko GR Wen J. Mitochondrial stress-activated cGAS-STING pathway inhibits thermogenic program and contributes to overnutrition-induced obesity in mice. Commun Biol. (2020) 3:257. doi: 10.1038/s42003-020-0986-1, PMID: 32444826 PMC7244732

[5] Ma XM Geng K Law BY-K Wang P Pu YL Chen Q. Lipotoxicity-induced mtDNA release promotes diabetic cardiomyopathy by activating the cGAS-STING pathway in obesity-related diabetes. Cell Biol Toxicol. (2023) 39:277–99. doi: 10.1007/s10565-021-09692-z, PMID: 35235096 PMC10042943

[6] Sanz-Martos AB Roca M Plaza A Merino B Ruiz-Gayo M Olmo ND. Long-term saturated fat-enriched diets impair hippocampal learning and memory processes in a sex-dependent manner. Neuropharmacology. (2024) 259:110108. doi: 10.1016/j.neuropharm.2024.110108, PMID: 39128582

[7] Gao H Luo Z Ji Y Tang K Jin Z Ly C. Accumulation of microbial DNAs promotes to islet inflammation and β cell abnormalities in obesity in mice. Nat Commun. (2022) 13:565. doi: 10.1038/s41467-022-28239-2, PMID: 35091566 PMC8799656

[8] Bhat N Mani A. Dysregulation of lipid and glucose metabolism in nonalcoholic fatty liver disease. Nutrients. (2023) 15:2323. doi: 10.3390/nu15102323, PMID: 37242206 PMC10222271

[9] Luo X Li H Ma L Zhou J Guo X Woo S-L. Expression of STING is increased in liver tissues from patients with NAFLD and promotes macrophage-mediated hepatic inflammation and fibrosis in mice. Gastroenterology. (2018) 155:1971–1984.e4. doi: 10.1053/j.gastro.2018.09.010, PMID: 30213555 PMC6279491

[10] Hu D Cui Y-X Wu M-Y Li L Su L-N Lian Z. Cytosolic DNA sensor cGAS plays an essential pathogenetic role in pressure overload-induced heart failure. Am J Phys Heart Circ Phys. (2020) 318:H1525–37. doi: 10.1152/ajpheart.00097.2020 32383996

[11] Xiong X Wang Q Wang S Zhang J Liu T Guo L. Mapping the molecular signatures of diet-induced NASH and its regulation by the hepatokine Tsukushi. Mol Metab. (2019) 20:128–37. doi: 10.1016/j.molmet.2018.12.004, PMID: 30595550 PMC6358550

[12] Li T Chen ZJ. The cGAS-cGAMP-STING pathway connects DNA damage to inflammation, senescence, and cancer. J Exp Med. (2018) 215:1287–99. doi: 10.1084/jem.20180139, PMID: 29622565 PMC5940270

[13] Bai J Liu F. The cGAS-cGAMP-STING pathway: a molecular link between immunity and metabolism. Diabetes. (2019) 68:1099–108. doi: 10.2337/dbi18-0052, PMID: 31109939 PMC6610018

[14] Gekara NO Jiang H. The innate immune DNA sensor cGAS: a membrane, cytosolic, or nuclear protein? Sci Signal. (2019) 12:eaax3521. doi: 10.1126/scisignal.aax3521 31088977

[15] Zierhut C Yamaguchi N Paredes M Luo J-D Carroll T Funabiki H. The cytoplasmic DNA sensor cGAS promotes mitotic cell death. Cell. (2019) 178:302–315.e23. doi: 10.1016/j.cell.2019.05.035, PMID: 31299200 PMC6693521

[16] Boyer JA Spangler CJ Strauss JD Cesmat AP Liu P McGinty RK. Structural basis of nucleosome-dependent cGAS inhibition. Science. (2020) 370:450–4. doi: 10.1126/science.abd0609 32913000 PMC8189757

[17] Barnett KC Coronas-Serna JM Zhou W Ernandes MJ Cao A Kranzusch PJ. Phosphoinositide interactions position cGAS at the plasma membrane to ensure efficient distinction between self- and viral DNA. Cell. (2019) 176:1432–1446.e11. doi: 10.1016/j.cell.2019.01.049, PMID: 30827685 PMC6697112

[18] Song B Greco TM Lum KK Taber CE Cristea IM. The DNA sensor cGAS is decorated by acetylation and phosphorylation modifications in the context of immune signaling. Mol Cell Proteomics. (2020) 19:1193–208. doi: 10.1074/mcp.RA120.001981, PMID: 32345711 PMC7338091

[19] Zhong L Hu M-M Bian L-J Liu Y Chen Q Shu H-B. Phosphorylation of cGAS by CDK1 impairs self-DNA sensing in mitosis. Cell Discov. (2020) 6:26. doi: 10.1038/s41421-020-0162-2, PMID: 32351706 PMC7186227

[20] Seo GJ Yang A Tan B Kim S Liang Q Choi Y. Akt kinase-mediated checkpoint of cGAS DNA sensing pathway. Cell Rep. (2015) 13:440–9. doi: 10.1016/j.celrep.2015.09.007, PMID: 26440888 PMC4607670

[21] Deribe YL Pawson T Dikic I. Post-translational modifications in signal integration. Nat Struct Mol Biol. (2010) 17:666–72. doi: 10.1038/nsmb.1842 20495563

[22] Wang Z Chen N Li Z Xu G Zhan X Tang J. The cytosolic DNA-sensing cGAS-STING pathway in liver diseases. Front Cell Dev Biol. (2021) 9:717610. doi: 10.3389/fcell.2021.717610 34386500 PMC8353273

[23] Sun X Liu T Zhao J Xia H Xie J Guo Y. DNA-PK deficiency potentiates cGAS-mediated antiviral innate immunity. Nat Commun. (2020) 11:6182. doi: 10.1038/s41467-020-19941-0, PMID: 33273464 PMC7712783

[24] Li H Hu L Wang L Wang Y Shao M Chen Y. Iron activates cGAS-STING signaling and promotes hepatic inflammation. J Agric Food Chem. (2022) 70:2211–20. doi: 10.1021/acs.jafc.1c06681, PMID: 35133148

[25] Zhong B Yang Y Li S Wang Y-Y Li Y Diao F. The adaptor protein MITA links virus-sensing receptors to IRF3 transcription factor activation. Immunity. (2008) 29:538–50. doi: 10.1016/j.immuni.2008.09.003, PMID: 18818105

[26] Gao P Hu M-M Shu H-B. CSK promotes innate immune response to DNA virus by phosphorylating MITA. Biochem Biophys Res Commun. (2020) 526:199–205. doi: 10.1016/j.bbrc.2020.03.069 32201077

[27] Ni G Ma Z Wong Jason P Zhang Z Cousins E Major MB. PPP6C negatively regulates STING-dependent innate immune responses. mBio. (2020) 11:e01728. doi: 10.1128/mBio.01728-20 32753499 PMC7407089

[28] Wang C Sharma N Veleeparambil M Kessler Patricia M Willard B Sen Ganes C. STING-mediated interferon induction by herpes simplex virus 1 requires the protein tyrosine kinase Syk. mBio. (2021) 12:e0322821. doi: 10.1128/mbio.03228-21 34933455 PMC8689565

[29] Wang C Wang X Veleeparambil M Kessler PM Willard B Chattopadhyay S. EGFR‐mediated tyrosine phosphorylation of STING determines its trafficking route and cellular innate immunity functions. EMBO J. (2020) 39:e104106. doi: 10.15252/embj.2019104106, PMID: 32926474 PMC7667877

[30] Gao L Zhang W Shi X Chang X Han Y Liu C. The mechanism of linear ubiquitination in regulating cell death and correlative diseases. Cell Death Dis. (2023) 14:659. doi: 10.1038/s41419-023-06183-3, PMID: 37813853 PMC10562472

[31] Liu W Tang X Qi X Fu X Ghimire S Ma R. The ubiquitin conjugating enzyme: an important ubiquitin transfer platform in ubiquitin-proteasome system. Int J Mol Sci. (2020) 21:2894. doi: 10.3390/ijms21082894 32326224 PMC7215765

[32] Sun M Zhang X. Current methodologies in protein ubiquitination characterization: from ubiquitinated protein to ubiquitin chain architecture. Cell Biosci. (2022) 12:126. doi: 10.1186/s13578-022-00870-y, PMID: 35962460 PMC9373315

[33] Zhang J Wang B Gao X Peng C Shan C Johnson SF. RNF185 regulates proteostasis in ebolavirus infection by crosstalk between the calnexin cycle, ERAD, and reticulophagy. Nat Commun. (2022) 13:6007. doi: 10.1038/s41467-022-33805-9, PMID: 36224200 PMC9554868

[34] Wang Q Huang L Hong Z Lv Z Mao Z Tang Y. The E3 ubiquitin ligase RNF185 facilitates the cGAS-mediated innate immune response. PLoS Pathog. (2017) 13:e1006264. doi: 10.1371/journal.ppat.1006264, PMID: 28273161 PMC5358892

[35] Liu Z-S Zhang Z-Y Cai H Zhao M Mao J Dai J. RINCK-mediated monoubiquitination of cGAS promotes antiviral innate immune responses. Cell Biosci. (2018) 8:35. doi: 10.1186/s13578-018-0233-3 29760876 PMC5944131

[36] Chen X Chen Y. Ubiquitination of cGAS by TRAF6 regulates anti-DNA viral innate immune responses. Biochem Biophys Res Commun. (2019) 514:659–64. doi: 10.1016/j.bbrc.2019.05.022 31078259

[37] Zhang W Li G Zhou X Liang H Tong B Wu D. Disassembly of the TRIM56-ATR complex promotes cytoDNA/cGAS/STING axis-dependent intervertebral disc inflammatory degeneration. J Clin Invest. (2024) 134:e165140. doi: 10.1172/JCI165140, PMID: 38488012 PMC10940101

[38] Fu L Zhou X Jiao Q Chen X. The functions of TRIM56 in antiviral innate immunity and tumorigenesis. Int J Mol Sci. (2023) 24:5046. doi: 10.3390/ijms24055046 36902478 PMC10003129

[39] Joshi B Joshi JC Mehta D. Regulation of cGAS activity and downstream signaling. Cells. (2022) 11:2812. doi: 10.3390/cells11182812 36139387 PMC9496985

[40] Li D Wu R Guo W Xie L Qiao Z Chen S. STING-mediated IFI16 degradation negatively controls type I interferon production. Cell Rep. (2019) 29:1249–1260.e4. doi: 10.1016/j.celrep.2019.09.069, PMID: 31665637

[41] Sun L Wu J Du F Chen X Chen ZJ. Cyclic GMP-AMP synthase is a cytosolic DNA sensor that activates the type I interferon pathway. Science. (2013) 339:786–91. doi: 10.1126/science.1232458, PMID: 23258413 PMC3863629

[42] McGuckin Wuertz K Treuting PM Hemann EA Esser-Nobis K Snyder AG Graham JB. STING is required for host defense against neuropathological West Nile virus infection. PLoS Pathog. (2019) 15:e1007899. doi: 10.1371/journal.ppat.1007899, PMID: 31415679 PMC6695101

[43] Liu N Pang X Zhang H Ji P. The cGAS-STING pathway in bacterial infection and bacterial immunity. Front Immunol. (2022) 12:814709. doi: 10.3389/fimmu.2021.814709 35095914 PMC8793285

[44] Aarreberg LD Esser-Nobis K Driscoll C Shuvarikov A Roby JA Gale M. Interleukin-1β induces mtDNA release to activate innate immune signaling via cGAS-STING. Mol Cell. (2019) 74:801–815.e6. doi: 10.1016/j.molcel.2019.02.038, PMID: 30952515 PMC6596306

[45] Aguirre S Fernandez-Sesma A. Collateral damage during dengue virus infection: making sense of DNA by cGAS. J Virol. (2017) 91:e01081. doi: 10.1128/JVI.01081-16 PMC5487551 28446670

[46] An J Durcan L Karr RM Briggs TA Rice GI Teal TH. Expression of cyclic GMP-AMP synthase in patients with systemic lupus erythematosus. Arthritis Rheumatol. (2017) 69:800–7. doi: 10.1002/art.40002, PMID: 27863149

[47] Sliter DA Martinez J Hao L Chen X Sun N Fischer TD. Parkin and PINK1 mitigate STING-induced inflammation. Nature. (2018) 561:258–62. doi: 10.1038/s41586-018-0448-9, PMID: 30135585 PMC7362342

[48] Jiang M Chen P Wang L Li W Chen B Liu Y. cGAS-STING, an important pathway in cancer immunotherapy. J Hematol Oncol. (2020) 13:81. doi: 10.1186/s13045-020-00916-z, PMID: 32571374 PMC7310007

[49] Corrales L McWhirter SM Dubensky TW Jr Gajewski TF. The host STING pathway at the interface of cancer and immunity. J Clin Invest. (2016) 126:2404–11. doi: 10.1172/JCI86892, PMID: 27367184 PMC4922692

[50] Flood BA Higgs EF Li S Luke JJ Gajewski TF. STING pathway agonism as a cancer therapeutic. Immunol Rev. (2019) 290:24–38. doi: 10.1111/imr.12765, PMID: 31355488 PMC6814203

[51] Yu Y Liu Y An W Song J Zhang Y Zhao X. STING-mediated inflammation in Kupffer cells contributes to progression of nonalcoholic steatohepatitis. J Clin Invest. (2019) 129:546–55. doi: 10.1172/JCI121842, PMID: 30561388 PMC6355218

[52] Kerur N Fukuda S Banerjee D. cGAS drives noncanonical-inflammasome activation in age-related macular degeneration. Nat Med. (2018) 24:50–61. doi: 10.1038/nm.4450, PMID: 29176737 PMC5760363

[53] Bodda C Reinert LS Fruhwürth S Richardo T Sun C Zhang B-C. HSV1 VP1-2 deubiquitinates STING to block type I interferon expression and promote brain infection. J Exp Med. (2020) 217:e20191422. doi: 10.1084/jem.20191422, PMID: 32383759 PMC7336311

[54] Zhang Q Jia Q Gao W Zhang W. The role of deubiquitinases in virus replication and host innate immune response. Front Microbiol. (2022) 13:13. doi: 10.3389/fmicb.2022.839624 PMC8908266 35283827

[55] Ventura-Clapier R Piquereau J Garnier A Mericskay M Lemaire C Crozatier B. Gender issues in cardiovascular diseases. Focus on energy metabolism. Biochim Biophys Acta Mol Basis Dis. (2020) 1866:165722. doi: 10.1016/j.bbadis.2020.165722, PMID: 32057941

[56] Geraghty L Figtree GA Schutte AE Patel S Woodward M Arnott C. Cardiovascular disease in women: from pathophysiology to novel and emerging risk factors. Heart Lung Circ. (2021) 30:9–17. doi: 10.1016/j.hlc.2020.05.108, PMID: 32843293

[57] Guo J Huang X Dou L Yan M Shen T Tang W. Aging and aging-related diseases: from molecular mechanisms to interventions and treatments. Signal Transduct Target Ther. (2022) 7:391. doi: 10.1038/s41392-022-01251-0, PMID: 36522308 PMC9755275

[58] Gusev E Sarapultsev A. Atherosclerosis and inflammation: insights from the theory of general pathological processes. Int J Mol Sci. (2023) 24:7910. doi: 10.3390/ijms24097910 37175617 PMC10178362

[59] Amen OM Sarker SD Ghildyal R Arya A. Endoplasmic reticulum stress activates unfolded protein response signaling and mediates inflammation, obesity, and cardiac dysfunction: therapeutic and molecular approach. Front Pharmacol. (2019) 10:977. doi: 10.3389/fphar.2019.00977, PMID: 31551782 PMC6747043

[60] Fricker M Tolkovsky AM Borutaite V Coleman M Brown GC. Neuronal cell death. Physiol Rev. (2018) 98:813–80. doi: 10.1152/physrev.00011.2017, PMID: 29488822 PMC5966715

[61] Fang L Ying S Xu X Wu D. TREX1 cytosolic DNA degradation correlates with autoimmune disease and cancer immunity. Clin Exp Immunol. (2023) 211:193–207. doi: 10.1093/cei/uxad017, PMID: 36745566 PMC10038326

[62] Morita M Stamp G Robins P Dulic A Rosewell I Hrivnak G. Gene-targeted mice lacking the Trex1 (DNase III) 3′→5′ DNA exonuclease develop inflammatory myocarditis. Mol Cell Biol. (2004) 24:6719–27. doi: 10.1128/MCB.24.15.6719-6727.2004, PMID: 15254239 PMC444847

[63] Gray EE Treuting PM Woodward JJ Stetson DB. Cutting edge: cGAS is required for lethal autoimmune disease in the Trex1-deficient mouse model of Aicardi–Goutières syndrome. J Immunol. (2015) 195:1939–43. doi: 10.4049/jimmunol.1500969, PMID: 26223655 PMC4546858

[64] An C Li Z Chen Y Huang S Yang F Hu Y. The cGAS-STING pathway in cardiovascular diseases: from basic research to clinical perspectives. Cell Biosci. (2024) 14:58. doi: 10.1186/s13578-024-01242-4, PMID: 38720328 PMC11080250

[65] King KR Aguirre AD Ye Y-X Sun Y Roh JD Ng RP. IRF3 and type I interferons fuel a fatal response to myocardial infarction. Nat Med. (2017) 23:1481–7. doi: 10.1038/nm.4428, PMID: 29106401 PMC6477926

[66] Luo W Wang Y Zhang L Ren P Zhang C Li Y. Critical role of cytosolic DNA and its sensing adaptor STING in aortic degeneration, dissection, and rupture. Circulation. (2020) 141:42–66. doi: 10.1161/CIRCULATIONAHA.119.041460, PMID: 31887080 PMC6939474

[67] Liu H Cheng W-L Jiang X Wang P-X Fang C Zhu X-Y. Ablation of interferon regulatory factor 3 protects against atherosclerosis in apolipoprotein E–deficient mice. Hypertension. (2017) 69:510–20. doi: 10.1161/HYPERTENSIONAHA.116.08395, PMID: 28115514

[68] Kong P Cui Z-Y Huang X-F Zhang D-D Guo R-J Han M. Inflammation and atherosclerosis: signaling pathways and therapeutic intervention. Signal Transduct Target Ther. (2022) 7:131. doi: 10.1038/s41392-022-00955-7, PMID: 35459215 PMC9033871

[69] Farahi L Sinha SK Lusis AJ. Roles of macrophages in atherogenesis. Front Pharmacol. (2021) 12:785220. doi: 10.3389/fphar.2021.785220 34899348 PMC8660976

[70] Bi X Du C Wang X Wang X-Y Han W Wang Y. Mitochondrial damage-induced innate immune activation in vascular smooth muscle cells promotes chronic kidney disease-associated plaque vulnerability. Adv Sci. (2021) 8:2002738. doi: 10.1002/advs.202002738, PMID: 33717842 PMC7927614

[71] Huang LS Hong Z Wu W Xiong S Zhong M Gao X. mtDNA activates cGAS signaling and suppresses the YAP-mediated endothelial cell proliferation program to promote inflammatory injury. Immunity. (2020) 52:475–486.e5. doi: 10.1016/j.immuni.2020.02.002, PMID: 32164878 PMC7266657

[72] Newman LE Shadel GS. Mitochondrial DNA release in innate immune signaling. Annu Rev Biochem. (2023) 92:299–332. doi: 10.1146/annurev-biochem-032620-104401 37001140 PMC11058562

[73] Cao Y Chen X Zhu Z Luo Z Hao Y Yang X. STING contributes to lipopolysaccharide-induced tubular cell inflammation and pyroptosis by activating endoplasmic reticulum stress in acute kidney injury. Cell Death Dis. (2024) 15:217. doi: 10.1038/s41419-024-06600-1, PMID: 38485717 PMC10940292

[74] Oduro PK Zheng X Wei J Yang Y Wang Y Zhang H. The cGAS-STING signaling in cardiovascular and metabolic diseases: future novel target option for pharmacotherapy. Acta Pharm Sin B. (2022) 12:50–75. doi: 10.1016/j.apsb.2021.05.011, PMID: 35127372 PMC8799861

[75] Gao J Cao H Hu G Wu Y Xu Y Cui H. The mechanism and therapy of aortic aneurysms. Signal Transduct Target Ther. (2023) 8:55. doi: 10.1038/s41392-023-01325-7 36737432 PMC9898314

[76] Rombouts KB van Merrienboer TAR Ket JCF Bogunovic N van der Velden J Yeung KK. The role of vascular smooth muscle cells in the development of aortic aneurysms and dissections. Eur J Clin Investig. (2022) 52:e13697. doi: 10.1111/eci.13697, PMID: 34698377 PMC9285394

[77] Ganizada BH Veltrop RJA Akbulut AC Koenen RR Accord R Lorusso R. Unveiling cellular and molecular aspects of ascending thoracic aortic aneurysms and dissections. Basic Res Cardiol. (2024) 119:371–95. doi: 10.1007/s00395-024-01053-1, PMID: 38700707 PMC11143007

[78] Bao T Liu J Leng J Cai L. The cGAS-STING pathway: more than fighting against viruses and cancer. Cell Biosci. (2021) 11:209. doi: 10.1186/s13578-021-00724-z, PMID: 34906241 PMC8670263

[79] Andrade B Jara-Gutiérrez C Paz-Araos M Vázquez MC Díaz P Murgas P. The relationship between reactive oxygen species and the cGAS/STING signaling pathway in the Inflammaging process. Int J Mol Sci. (2022) 23:15182. doi: 10.3390/ijms232315182, PMID: 36499506 PMC9735967

[80] Daoud F Arévalo Martínez M Holst J Holmberg J Albinsson S Swärd K. Role of smooth muscle YAP and TAZ in protection against phenotypic modulation, inflammation, and aneurysm development. Biochem Pharmacol. (2022) 206:115307. doi: 10.1016/j.bcp.2022.115307, PMID: 36270325

[81] Liu S Guan W. STING signaling promotes apoptosis, necrosis, and cell death: an overview and update. Mediat Inflamm. (2018) 2018:1202797. doi: 10.1155/2018/1202797 PMC6286756 30595664

[82] Zhang Y Chen W Wang Y. STING is an essential regulator of heart inflammation and fibrosis in mice with pathological cardiac hypertrophy via endoplasmic reticulum (ER) stress. Biomed Pharmacother. (2020) 125:110022. doi: 10.1016/j.biopha.2020.110022, PMID: 32106379

[83] Wang S Wang L Qin X Turdi S Sun D Culver B. ALDH2 contributes to melatonin-induced protection against APP/PS1 mutation-prompted cardiac anomalies through cGAS-STING-TBK1-mediated regulation of mitophagy. Signal Transduct Target Ther. (2020) 5:119. doi: 10.1038/s41392-020-0171-5, PMID: 32703954 PMC7378833

[84] Maekawa H Inoue T Ouchi H Jao T-M Inoue R Nishi H. Mitochondrial damage causes inflammation via cGAS-STING signaling in acute kidney injury. Cell Rep. (2019) 29:1261–1273.e6. doi: 10.1016/j.celrep.2019.09.050, PMID: 31665638

[85] Bai J Liu F. cGAS-STING signaling and function in metabolism and kidney diseases. J Mol Cell Biol. (2021) 13:728–38. doi: 10.1093/jmcb/mjab066, PMID: 34665236 PMC8718186

[86] Guo Y Gu R Gan D Hu F Li G Xu G. Mitochondrial DNA drives noncanonical inflammation activation via cGAS-STING signaling pathway in retinal microvascular endothelial cells. Cell Commun Signal. (2020) 18:172. doi: 10.1186/s12964-020-00637-3, PMID: 33115500 PMC7592595

[87] He W Mu X Wu X Liu Y Deng J Liu Y. The cGAS-STING pathway: a therapeutic target in diabetes and its complications. Burns Trauma. (2024):12. doi: 10.1016/j.ijbiomac.2024.134210 PMC10838060 38312740

[88] Zong Y Li H Liao P Chen L Pan Y Zheng Y. Mitochondrial dysfunction: mechanisms and advances in therapy. Signal Transduct Target Ther. (2024) 9:124. doi: 10.1038/s41392-024-01839-8, PMID: 38744846 PMC11094169

[89] Odegaard JI Chawla A. Type 2 responses at the interface between immunity and fat metabolism. Curr Opin Immunol. (2015) 36:67–72. doi: 10.1016/j.coi.2015.07.003, PMID: 26204571 PMC4593747

[90] Alwarawrah Y Kiernan K MacIver NJ. Changes in nutritional status impact immune cell metabolism and function. Front Immunol. (2018) 9:1055. doi: 10.3389/fimmu.2018.01055 29868016 PMC5968375

[91] Kawai T Autieri MV Scalia R. Adipose tissue inflammation and metabolic dysfunction in obesity. Am J Phys Cell Phys. (2020) 320:C375–91. doi: 10.1152/ajpcell.00379.2020, PMID: 33356944 PMC8294624

[92] Esser N Legrand-Poels S Piette J Scheen AJ Paquot N. Inflammation as a link between obesity, metabolic syndrome and type 2 diabetes. Diabetes Res Clin Pract. (2014) 105:141–50. doi: 10.1016/j.diabres.2014.04.006 24798950

[93] Wang T He C. Pro-inflammatory cytokines: the link between obesity and osteoarthritis. Cytokine Growth Factor Rev. (2018) 44:38–50. doi: 10.1016/j.cytogfr.2018.10.002, PMID: 30340925

[94] Patel SJ Liu N Piaker S Gulko A Andrade ML Heyward FD. Hepatic IRF3 fuels dysglycemia in obesity through direct regulation of *Ppp2r1b*. Sci Transl Med. (2022) 14:eabh3831. doi: 10.1126/scitranslmed.abh3831 35320000 PMC9162056

[95] Tang P Virtue S Goie JYG Png CW Guo J Li Y. Regulation of adipogenic differentiation and adipose tissue inflammation by interferon regulatory factor 3. Cell Death Differ. (2021) 28:3022–35. doi: 10.1038/s41418-021-00798-9, PMID: 34091599 PMC8563729

[96] Wang X-A Zhang R She Z-G Zhang X-F Jiang D-S Wang T. Interferon regulatory factor 3 constrains IKKβ/NF-κB signaling to alleviate hepatic steatosis and insulin resistance. Hepatology. (2014) 59:870–85. doi: 10.1002/hep.26751, PMID: 24123166

[97] Kumari M Wang X Lantier L Lyubetskaya A Eguchi J Kang S. IRF3 promotes adipose inflammation and insulin resistance and represses browning. J Clin Invest. (2016) 126:2839–54. doi: 10.1172/JCI86080, PMID: 27400129 PMC4966307

[98] Muñoz MC Giani JF Mayer MA Toblli JE Turyn D Dominici FP. TANK-binding kinase 1 mediates phosphorylation of insulin receptor at serine residue 994: a potential link between inflammation and insulin resistance. J Endocrinol. (2009) 201:185–97. doi: 10.1677/JOE-08-0276, PMID: 19251743

[99] Cruz VH Arner EN Wynne KW Scherer PE Brekken RA. Loss of Tbk1 kinase activity protects mice from diet-induced metabolic dysfunction. Mol Metab. (2018) 16:139–49. doi: 10.1016/j.molmet.2018.06.007, PMID: 29935921 PMC6157474

[100] Bodur C Kazyken D Huang K Tooley AS Cho KW Barnes TM. TBK1-mTOR signaling attenuates obesity-linked hyperglycemia and insulin resistance. Diabetes. (2022) 71:2297–312. doi: 10.2337/db22-0256, PMID: 35983955 PMC9630091

[101] Zhao P Ki W Sun X Reilly SM Uhm M Liao Z. TBK1 at the crossroads of inflammation and energy homeostasis in adipose tissue. Cell. (2018) 172:731–743.e12. doi: 10.1016/j.cell.2018.01.007 29425491 PMC5808582

[102] Reilly SM Chiang S-H Decker SJ Chang L Uhm M Larsen MJ. An inhibitor of the protein kinases TBK1 and IKK-ɛ improves obesity-related metabolic dysfunctions in mice. Nat Med. (2013) 19:313–21. doi: 10.1038/nm.3082, PMID: 23396211 PMC3594079

[103] Yan S Kumari M Xiao H Jacobs C Kochumon S Jedrychowski M. IRF3 reduces adipose thermogenesis via ISG15-mediated reprogramming of glycolysis. J Clin Invest. (2021) 131:e144888. doi: 10.1172/JCI144888, PMID: 33571167 PMC8011904

[104] Madison BB. Srebp2: A master regulator of sterol and fatty acid synthesis. J Lipid Res. (2016) 57:333–5. doi: 10.1194/jlr.C066712, PMID: 26798145 PMC4766982

[105] Shimano H Sato R. SREBP-regulated lipid metabolism: convergent physiology—divergent pathophysiology. Nat Rev Endocrinol. (2017) 13:710–30. doi: 10.1038/nrendo.2017.91, PMID: 28849786

[106] York Autumn G Williams Kevin J Argus Joseph P Zhou Quan D Brar G Vergnes L. Limiting cholesterol biosynthetic flux spontaneously engages type I IFN signaling. Cell. (2015) 163:1716–29. doi: 10.1016/j.cell.2015.11.045, PMID: 26686653 PMC4783382

[107] Chen W Li S Yu H Liu X Huang L Wang Q. ER adaptor SCAP translocates and recruits IRF3 to perinuclear microsome induced by cytosolic microbial DNAs. PLoS Pathog. (2016) 12:e1005462. doi: 10.1371/journal.ppat.1005462, PMID: 26900919 PMC4762662

[108] Griffiths WJ Wang Y. Cholesterol metabolism: from lipidomics to immunology. J Lipid Res. (2022) 63:100165. doi: 10.1016/j.jlr.2021.100165, PMID: 34953867 PMC8953665

[109] Lv J Xing C Chen Y Bian H Lv N Wang Z. The STING in non-alcoholic fatty liver diseases: potential therapeutic targets in inflammation-carcinogenesis pathway. Pharmaceuticals. (2022) 15:1241. doi: 10.3390/ph15101241 36297353 PMC9611148

[110] Chu T-T Tu X Yang K Wu J Repa JJ Yan N. Tonic prime-boost of STING signalling mediates Niemann–Pick disease type C. Nature. (2021) 596:570–5. doi: 10.1038/s41586-021-03762-2, PMID: 34290407 PMC8859990

[111] Field CS Baixauli F Kyle RL Puleston DJ Cameron AM Sanin DE. Mitochondrial integrity regulated by lipid metabolism is a cell-intrinsic checkpoint for Treg suppressive function. Cell Metab. (2020) 31:422–437.e5. doi: 10.1016/j.cmet.2019.11.021, PMID: 31883840 PMC7001036

[112] Vos DY van de Sluis B. Function of the endolysosomal network in cholesterol homeostasis and metabolic-associated fatty liver disease (MAFLD). Mol Metab. (2021) 50:101146. doi: 10.1016/j.molmet.2020.101146, PMID: 33348067 PMC8324686

[113] Anstee QM Reeves HL Kotsiliti E Govaere O Heikenwalder M. From NASH to HCC: current concepts and future challenges. Nat Rev Gastroenterol Hepatol. (2019) 16:411–28. doi: 10.1038/s41575-019-0145-7, PMID: 31028350

[114] Wang X Rao H Zhao J Wee A Li X Fei R. STING expression in monocyte-derived macrophages is associated with the progression of liver inflammation and fibrosis in patients with nonalcoholic fatty liver disease. Lab Investig. (2020) 100:542–52. doi: 10.1038/s41374-019-0342-6 31745210

[115] Qiao JT Cui C Qing L Wang LS He TY Yan F. Activation of the STING-IRF3 pathway promotes hepatocyte inflammation, apoptosis and induces metabolic disorders in nonalcoholic fatty liver disease. Metab Clin Exp. (2018) 81:13–24. doi: 10.1016/j.metabol.2017.09.010, PMID: 29106945

[116] Donne R Saroul-Ainama M Cordier P Hammoutene A Kabore C Stadler M. Replication stress triggered by nucleotide pool imbalance drives DNA damage and cGAS-STING pathway activation in NAFLD. Dev Cell. (2022) 57:1728–1741.e6. doi: 10.1016/j.devcel.2022.06.003, PMID: 35768000

[117] Iracheta-Vellve A Petrasek J Gyongyosi B Satishchandran A Lowe P Kodys K. Endoplasmic reticulum stress-induced hepatocellular death pathways mediate liver injury and fibrosis via stimulator of interferon genes. J Biol Chem. (2016) 291:26794–805. doi: 10.1074/jbc.M116.736991, PMID: 27810900 PMC5207187

[118] Chen J Li J Yiu JHC Lam JKW Wong C-M Dorweiler B. TRIF-dependent Toll-like receptor signaling suppresses *Scd1* transcription in hepatocytes and prevents diet-induced hepatic steatosis. Sci Signal. (2017) 10:eaal3336. doi: 10.1126/scisignal.aal3336 28790196

[119] Guo X Shu C Li H Pei Y Woo S-L Zheng J. Cyclic GMP-AMP ameliorates diet-induced metabolic dysregulation and regulates proinflammatory responses distinctly from STING activation. Sci Rep. (2017) 7:6355. doi: 10.1038/s41598-017-05884-y 28743914 PMC5526935

[120] Cho CS Park HW Ho A Semple IA Kim B Jang I. Lipotoxicity induces hepatic protein inclusions through TANK binding kinase 1-mediated p62/sequestosome 1 phosphorylation. Hepatology. (2018) 68:1331–46. doi: 10.1002/hep.29742, PMID: 29251796 PMC6005718

[121] Petrasek J Iracheta-Vellve A Csak T Satishchandran A Kodys K Kurt-Jones EA. STING-IRF3 pathway links endoplasmic reticulum stress with hepatocyte apoptosis in early alcoholic liver disease. Proc Natl Acad Sci USA. (2013) 110:16544–9. doi: 10.1073/pnas.1308331110, PMID: 24052526 PMC3799324

[122] Luther J Khan S Gala MK Kedrin D Sridharan G Goodman RP. Hepatic gap junctions amplify alcohol liver injury by propagating cGAS-mediated IRF3 activation. Proc Natl Acad Sci USA. (2020) 117:11667–73. doi: 10.1073/pnas.1911870117, PMID: 32393626 PMC7261084

[123] Hong Z Mei J Li C Bai G Maimaiti M Hu H. STING inhibitors target the cyclic dinucleotide binding pocket. Proc Natl Acad Sci USA. (2021) 118:e2105465118. doi: 10.1073/pnas.2105465118, PMID: 34099558 PMC8214703

[124] Pan Y You Y Sun L Sui Q Liu L Yuan H. The STING antagonist H-151 ameliorates psoriasis via suppression of STING/NF-κB-mediated inflammation. Br J Pharmacol. (2021) 178:4907–22. doi: 10.1111/bph.15673, PMID: 34460100

[125] Zhang S Zheng R Pan Y Sun H. Potential therapeutic value of the STING inhibitors. Molecules. (2023) 28:3127. doi: 10.3390/molecules28073127, PMID: 37049889 PMC10096477

[126] Mullard A. Biotechs step on cGAS for autoimmune diseases. Nat Rev Drug Discov. (2023) 22:939–41. doi: 10.1038/d41573-023-00185-8, PMID: 37949966

[127] Wiser C Kim B Vincent J Ascano M. Small molecule inhibition of human cGAS reduces total cGAMP output and cytokine expression in cells. Sci Rep. (2020) 10:7604. doi: 10.1038/s41598-020-64348-y, PMID: 32371942 PMC7200739

[128] Lama L Adura C Xie W Tomita D Kamei T Kuryavyi V. Development of human cGAS-specific small-molecule inhibitors for repression of dsDNA-triggered interferon expression. Nat Commun. (2019) 10:2261. doi: 10.1038/s41467-019-08620-4, PMID: 31113940 PMC6529454

[129] Chang J Hou S Yan X Li W Xiao J. Discovery of novel STING inhibitors based on the structure of the mouse STING agonist DMXAA. Molecules. (2023) 28. doi: 10.3390/molecules28072906, PMID: 37049669 PMC10095708

[130] Daei Farshchi Adli A Jahanban-Esfahlan R Seidi K Samandari-Rad S Zarghami N. An overview on vadimezan (DMXAA): the vascular disrupting agent. Chem Biol Drug Des. (2018) 91:996–1006. doi: 10.1111/cbdd.13166, PMID: 29288534

[131] Graham PT Nowak AK Cornwall SMJ Larma I Nelson DJ. The STING agonist, DMXAA, reduces tumor vessels and enhances mesothelioma tumor antigen presentation yet blunts cytotoxic T cell function in a murine model. Front Immunol. (2022) 13:969678. doi: 10.3389/fimmu.2022.969678, PMID: 36466911 PMC9716460

[132] Oral EA Reilly SM Gomez AV Meral R Butz L Ajluni N. Inhibition of IKKɛ and TBK1 improves glucose control in a subset of patients with type 2 diabetes. Cell Metab. (2017) 26:157–170.e7. doi: 10.1016/j.cmet.2017.06.006, PMID: 28683283 PMC5663294

[133] De Gaetano A Solodka K Zanini G Selleri V Mattioli AV Nasi M. Molecular mechanisms of mtDNA-mediated inflammation. Cells. (2021) 10:2898. doi: 10.3390/cells10112898 34831121 PMC8616383

[134] Esteves AM Papaevangelou E Dasgupta P Galustian C. Combination of Interleukin-15 with a STING agonist, ADU-S100 analog: a potential immunotherapy for prostate cancer. Front Oncol. (2021) 11:621550. doi: 10.3389/fonc.2021.621550 33777767 PMC7988118

[135] Papaevangelou E Esteves AM Dasgupta P Galustian C. Cyto-IL-15 synergizes with the STING agonist ADU-S100 to eliminate prostate tumors and confer durable immunity in mouse models. Front Immunol. (2023) 14:1196829. doi: 10.3389/fimmu.2023.1196829, PMID: 37465665 PMC10350564

[136] Liu K Lan Y Li X Li M Cui L Luo H. Development of small molecule inhibitors/agonists targeting STING for disease. Biomed Pharmacother. (2020) 132:110945. doi: 10.1016/j.biopha.2020.110945 33254439

[137] Maekawa H Fain ME Wasano K. Pathophysiological roles of the cGAS-STING inflammatory pathway. Physiology. (2023) 38:167–77. doi: 10.1152/physiol.00031.2022, PMID: 36976098

